# Soils and sediments host Thermoplasmata archaea encoding novel copper membrane monooxygenases (CuMMOs)

**DOI:** 10.1038/s41396-021-01177-5

**Published:** 2022-01-05

**Authors:** Spencer Diamond, Adi Lavy, Alexander Crits-Christoph, Paula B. Matheus Carnevali, Allison Sharrar, Kenneth H. Williams, Jillian F. Banfield

**Affiliations:** 1 Innovative Genomics Institute, University of California, Berkeley, CA USA; 2 Department of Earth and Planetary Science, University of California, Berkeley, CA USA; 3 Department of Plant and Microbial Biology, University of California, Berkeley, CA USA; 4 Climate and Ecosystem Sciences Division, Lawrence Berkeley National Laboratory, Berkeley, CA USA; 5 Department of Environmental Science, Policy and Management, University of California, Berkeley, CA USA; 6 School of Earth Sciences, University of Melbourne, Melbourne, VIC Australia

**Keywords:** Environmental microbiology, Microbial ecology, Soil microbiology, Next-generation sequencing

## Abstract

Copper membrane monooxygenases (CuMMOs) play critical roles in the global carbon and nitrogen cycles. Organisms harboring these enzymes perform the first, and rate limiting, step in aerobic oxidation of ammonia, methane, or other simple hydrocarbons. Within archaea, only organisms in the order Nitrososphaerales (Thaumarchaeota) encode CuMMOs, which function exclusively as ammonia monooxygenases. From grassland and hillslope soils and aquifer sediments, we identified 20 genomes from distinct archaeal species encoding divergent CuMMO sequences. These archaea are phylogenetically clustered in a previously unnamed Thermoplasmatota order, herein named the Ca. Angelarchaeales. The CuMMO proteins in Ca. Angelarchaeales are more similar in structure to those in Nitrososphaerales than those of bacteria, and contain all functional residues required for general monooxygenase activity. Ca. Angelarchaeales genomes are significantly enriched in blue copper proteins (BCPs) relative to sibling lineages, including plastocyanin-like electron carriers and divergent nitrite reductase-like (*nirK*) 2-domain cupredoxin proteins co-located with electron transport machinery. Ca. Angelarchaeales also encode significant capacity for peptide/amino acid uptake and degradation and share numerous electron transport mechanisms with the Nitrososphaerales. Ca. Angelarchaeales are detected at high relative abundance in some of the environments where their genomes originated from. While the exact substrate specificities of the novel CuMMOs identified here have yet to be determined, activity on ammonia is possible given their metabolic and ecological context. The identification of an archaeal CuMMO outside of the Nitrososphaerales significantly expands the known diversity of CuMMO enzymes in archaea and suggests previously unaccounted organisms contribute to critical global nitrogen and/or carbon cycling functions.

## Introduction

Copper membrane monooxygenases (CuMMOs) are a family of phylogenetically diverse and ecologically widespread but highly conserved enzymes that function in the aerobic oxidation of ammonia, methane, and potentially other hydrocarbon substrates [1–3]. Organisms encoding CuMMOs are critically important in global carbon and nitrogen cycles [4, 5], as particulate methane monooxygenases attenuate atmospheric methane release [5], and ammonia monooxygenases (AMOs) can generate nitrous oxide as a byproduct [6]. While the diversity of CuMMOs has been expanded due to cultivation independent studies [7–9], CuMMOs have still only been identified in a few monophyletic clades of Bacteria and Archaea [1]. The distribution and functionality of CuMMOs in Archaea is particularly constrained. Archaeal CuMMOs have only been shown to participate in ammonia oxidation, and Nitrososphaerales (Thaumarchaeota) are the only identified group to date that encode AMOs [8]. These ammonia oxidizing archaea (AOA) occur across a wide array of natural environments [10], and are often far more abundant than ammonia oxidizing bacteria (AOB) [11], resulting in large contributions to the global nitrogen cycle [3, 12]. The identification of an additional archaeal lineage with the capability to oxidize either ammonia, methane, or hydrocarbons using a CuMMO would have major implications for the microbial turnover of nitrogen and carbon in the biosphere respectively.

The CuMMO is a membrane bound protein complex that consists of three core proteins *amoA*/*pmoA*, *amoB*/*pmoB*, and *amoC*/*pmoC*, that are typically encoded in an operon [1, 13]. In AOA, a fourth hypothetical protein, *amoX*, of unknown function is also present [13]. Despite their ecological importance, the difficulties in sustaining in vitro activity of CuMMO complexes as well as difficulties in protein structure resolution has led to inconsistent reports regarding the co-factors and substrate specificity controls for these enzymes. Indeed the substrate specificity of CuMMOs is promiscuous and purified enzymes have been demonstrated to have activity on methane, ammonia, and small hydrocarbons [14, 15]. However, in the organismal context, the substrate specificity is highly preferential to a single substrate, and occurs in a metabolic context of enzymes that support the processing of that specific substrate and its byproducts [3, 16].

In both AOA and AOB, ammonia oxidation proceeds initially by the conversion of ammonia to hydroxylamine and subsequent oxidation of hydroxylamine to nitrite [17]. While the AMO mediated oxidation of ammonia to hydroxylamine has been established in AOA [18], the lack of an identifiable hydroxylamine dehydrogenase (*Hao*) in Archaea has led to the proposal of at least two hypothetical routes from hydroxylamine to nitrite [17, 19]. It has been postulated that *Hao* activity in AOA instead relies on a suite of blue copper proteins (BCPs), which are numerous in AOA lineages [16, 17, 19, 20]. Plastocyanin-like electron transport proteins, divergent Cu-containing nitrite reductases (*nirK*), and other 1 and 2 domain cupredoxin-like proteins have been shown to have correlated transcriptional activity with AMO proteins [21], but a conclusive link to *Hao* activity is lacking.

Aerobic methane oxidation has not yet been observed in archaea, however in bacteria the oxidation is well studied and proceeds initially by the CuMMO mediated conversion of methane to methanol [22]. Subsequently methanol is oxidized by a dedicated methanol dehydrogenase to formaldehyde, for which specific enzymes are also lacking in archaeal genomes. Formaldehyde can then be directly oxidized further by dedicated enzymes or can be incorporated into central carbon metabolism by a variety of routes [23].

Identification of novel CuMMO complexes in microbes, and surveys of CuMMOs in natural environments, has primarily relied on primer based nucleic acid amplification of the *amoA*/*pmoA* subunit [24]. However, the requirement of sufficient sequence similarity between environmental sequences and known bacterial and archaeal *amoA*/*pmoA* subunits precludes the identification of completely novel sequence variants. Further, primer-based studies do not provide information about genome context, which can place CuMMOs in a metabolic context that supports a specific activity. Here, we applied sensitive homology-based methods to de novo reconstructed metagenome assembled genomes (MAGs) obtained from soils, aquifer sediment, and deep ocean environments to identify novel CuMMO variants and describe their genomic, phylogenetic and ecological contexts. This new group of soil- and aquifer-associated archaea, that encode a novel CuMMO variant, may perform functions previously linked to a phylogenetically narrow range of bacteria and archaea.

## Results

### Divergent CuMMOs identified in MAGs recovered from soil and sediment ecosystems

**Fig. 1:**
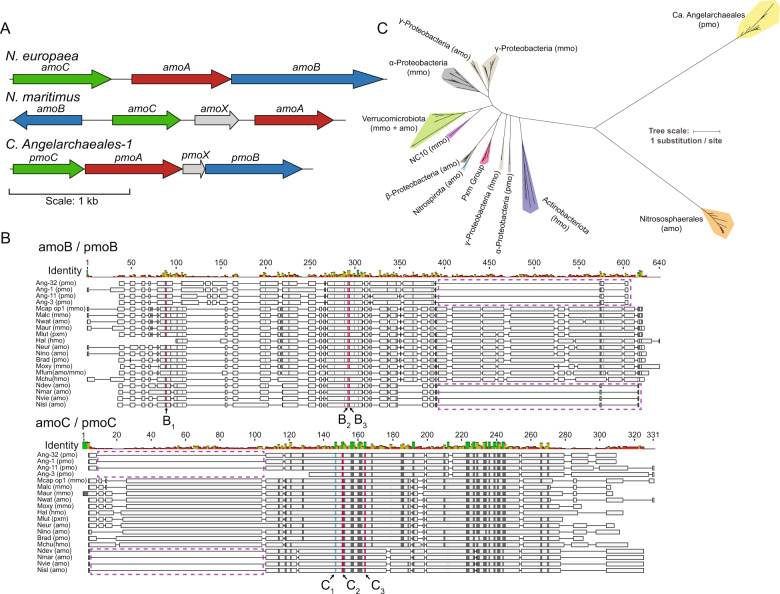
Comparison of divergent CuMMO proteins to known sequences. **A** Comparison of pmoABC loci and gene arrangement between Nitrosomonas europaea (ammonia oxidizing bacteria), Nitrosopumilus maritimus (ammonia oxidizing archaea), and Ca. Angelarchaeales-1. **B** Representative sequences from a multiple sequence alignment of amoB/pmoB proteins (top; *n* = 114 sequences) and amoC/pmoC proteins (bottom; *n* = 110 sequences). Sequences are named by species abbreviation followed by putative function in parentheses: pmo = particulate monooxygenase (no biochemical evidence), mmo = methane monooxygenase, amo = ammonia monooxygenase, hmo = hydrocarbon monooxygenase. Species abbreviations are described in Supplementary Table 3. Lettering below alignments indicates residues forming part of B or C-sites in these proteins. Histidine residues and aspartic acid residues within these sites are colored red and blue respectively. Residues colored black are identical across ≥ 65% of sequences in full alignment. Purple boxes indicate truncations shared by CuMMOs identified in this study and known archaeal sequences. All amoABC/pmoABC proteins and alignments are available in Supplementary Data 1. **C** Maximum likelihood phylogenetic tree constructed from a concatenated alignment of amoABC/pmoABC proteins (*n* = 112 sequences; ≥2 sequences per genome). Colored clades are drawn based on a combination of shared function and taxonomy and colored by the taxonomy of genomes encoding the sequences within the clade. Clades are labeled by their shared taxonomy and known/predicted CuMMO function in parentheses (see above). Pxm group is an exception and represents a group of duplicated proteins present in many gammaproteobacterial methanotrophs. Scale bar represents average changes per amino acid position.

In previous work we identified putative divergent *amoA*/*pmoA* homologues in 7 Thermoplasmatota genomes recovered from Mediterranean grassland soil [25]. This was intriguing, given that *amo*/*pmo* homologues had not been previously observed in archaea outside of the Nitrososphaerales. Here we searched for additional genomes encoding related (divergent) *amo*/*pmo*’s using a series of readily available, and custom built, hidden markov models (HMMs) across all archaeal genomes in the Genome Taxonomy Database (GTDB), and in all archaeal MAGs in our unpublished datasets from ongoing studies (Supplementary Fig. 1 and Supplementary Data 1). We found additional *amoA*/*pmoA* genes in genomes recovered from soils at the South Meadow and Rivendell sites of the Angelo Coast Range Reserve (CA) [25, 26], the nearby Sagehorn site [26], a hillslope of the East River watershed (CO) [27], and in sediments from the Rifle aquifer (CO) [28] and the deep ocean [29]. In total we identified 201 archaeal MAGs taxonomically placed using phylogenetically informative single copy marker genes outside of Nitrososphaerales containing divergent *amo*/*pmo* proteins (Supplementary Table 1 and Supplementary Data 1). Genome de-replication resulted in 34 species-level genome clusters, 20 of which encoded an *amo*/*pmo* homologue (Supplementary Table 2). Of these genomes, 11 are species not previously available in public databases. In all cases where assembled sequences were of sufficient length, the *amoA*/*pmoA*, B, and C protein coding genes were found co-located with each other and with a hypothetical protein here called *amoX*/*pmoX* in the order C-A-X-B (Fig. 1A, Supplementary Table 2, and Supplementary Fig. 2). The mean sequence identity of the novel *amoA*/*pmoA*, B, and C proteins to known bacterial sequences were 16.7, 8.0, and 14.2% and 13.8, 9.5, and 20.8% to known archaeal sequences. This level of divergent amino acid identity is typical for CuMMOs, as known bacterial and known archaeal *amoA*/*pmoA*, B, and C proteins share mean identities of 16.1, 9.7, and 16.5% respectively. As might be expected considering the large sequence divergence between the recovered sequences and known *amo*/*pmo* proteins, we found that no pair of typical primers used for bacterial and archaeal *amoA*/*pmoA* environmental surveys [30] matched any novel *amoA*/*pmoA* gene with <7 mismatched bases (Supplementary Table 3). This suggests that these sequences would have been missed by previous primer-based *amoA*/*pmoA* gene surveys.

### Novel CuMMO subunit sequences contain expected catalytic and metal binding residues

Alignments were constructed for each predicted *amoA*/*pmoA*, B, and C protein in combination with reference sequences that cover the known diversity of these protein subunits [1] (Fig. 1B and Supplementary Data 1). In the new sequences, all of the expected catalytic and metal binding residues known to be present in CuMMOs [31] were conserved (Fig. 1B and Supplementary Figs. 3, 4). In *amoB*/*pmoB*, all three histidine residues for the mono-copper B-site required for enzyme activity were conserved. The C-site in *amoC*/*pmoC*, which contains an aspartic acid and two histidines important for enzymatic activity [13], is also completely conserved. Although the A-sites in *amoB*/*pmoB* and the D-sites in *amoC*/*pmoC* and *amoA*/*pmoA* were not observed in the new sequences, these sites are not required for catalytic function and are only conserved within bacterial lineages [1, 8]. We also note that the new *amoB*/*pmoB* sequences share a C-terminal truncation to previously identified Nitrososphaerales *amoB*’s [32], as well as share an N-terminal truncation in the *amoC*/*pmoC* protein. As the proteins in these alignments shared very low sequence identity, we performed an assessment of alignment quality at each aligned position for the *amoB*/*pmoB* and *amoC*/*pmoC* alignments. We found that all active site positions in these alignments were positions aligned with high confidence (Supplementary Figs. 3, 4). Finally, we performed de novo structural prediction for a representative *amoB*/*pmoB* protein, as it is the putative catalytic subunit, using AlphaFold [33]. A structural similarity search against the Protein Data Bank (PDB) identified 4O65_A (cupredoxin domain of *amoB* from Nitrosocaldus yellowstonii) as the best matching structure to the *amoB*/*pmoB* protein model. Structural superposition of the *amoB*/*pmoB* model and 4O65_A structure showed proper alignment of the B_2_ and B_3_ site histidines (Supplementary Fig. 5 and Supplementary Data 1). The B_1_ site could not be analyzed as it was not included in the 4O65_A protein structure. We note that while the data strongly support the novel *amo*/*pmo* proteins identified here as genuine and active CuMMOs, the exact substrate specificity of these proteins cannot be conclusively determined from this data alone.

### Novel CuMMOs form a new group in the CuMMO superfamily

To infer the evolutionary relationship of our newly identified CuMMO sequences to known CuMMO family members we used a concatenated *amoABC*/*pmoABC* sequence alignment to produce a phylogenetic reconstruction covering known family members (Fig. 1C). Individual protein phylogenies were also constructed for each *pmo* subunit (Supplementary Fig. 6a–c). The different subunit reconstructions largely agree in overall topology, and all reconstructions support our newly identified sequences as a highly divergent third major lineage of CuMMOs. Also, similar to previous phylogenetic reconstructions of CuMMO sequences, our sequences form clusters that mirror the phylogenetic relatedness of encoding genomes [1].

### Archaea with divergent CuMMOs form a novel clade within the Thermoplasmatota

**Fig. 2:**
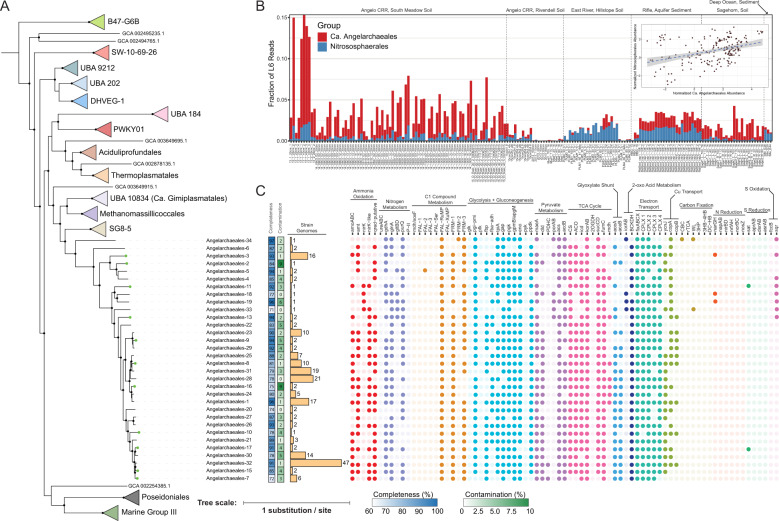
Taxonomy, metabolism, and distribution of Ca. Angelarchaeales. **A** Maximum likelihood phylogenetic tree constructed for the archaeal phylum Thermoplasmatota using a concatenated alignment of 122 archaeal specific marker genes. The tree includes 32 Ca. Angelarchaeales genomes and 281 reference genomes. The Ca. Lunaplasmatales lacustris genome is omitted from this tree as it was not metabolically analyzed in this work (See Supplementary Fig. 7). Tree was rooted using A. fulgidus (GCF_000008665.1) as an outgroup. Clades were collapsed at the order level if they contained more than one genome and arbitrarily colored. Black dots at nodes indicate ≥ 90% bootstrap support (ufboot; *n* = 1000). Green dots at leaf tips indicate genome from this study. For the full, un-collapsed, tree see Supplementary Fig. 7. **B** Relative abundance information, based on rpL6 read counts, for Ca. Angelarchaeales and Nitrososphaerales across 185 shotgun metagenome samples from six sites. The *x*-axis indicates the sample name, and the *y*-axis indicates the fraction of reads out of the total reads in a given sample that mapped to rpL6 sequences taxonomically associated with each group. Samples are separated by the general sampling location, indicated at the top of the plot. Inset, normalized rpL6 based relative abundance of Ca. Angelarchaeales (*x*-axis) vs. Nitrososphaerales (*y*-axis) for all 185 shotgun metagenome samples. A best fit line is plotted using linear regression, shaded area indicates standard error of the regression. Rho of association is positive and significant (rho = 0.366, FDR < 0.001). **C** Genome quality, number of strain-level genomes (genomes with ANI ≥ 95%), and predicted metabolism for the 32 Ca. Angelarchaeales genomes in (**A**). Filled dots indicate the presence of a gene or gene set that executes a specific metabolic function or reaction. Dots are colored based on shared pathways or metabolic functionality as described above the figure, and colors are chosen arbitrarily. For complete explanation of metabolic functions and search criteria see Supplementary Table 9.

An initial phylogenetic classification of our archaeal genomes placed them in a yet unnamed order-level lineage, RBG-16-68-12, within the Thermoplasmatota phylum (Supplementary Tables 1, 2). A concatenated alignment of 122 archaeal specific marker proteins [34] resolved them as a well-supported order-level monophyletic lineage basal to the candidate SG8-5 lineage within *Thermoplasmatota* (Fig. 2A, Supplementary Fig. 7, and Supplementary Table 4). We reported the first genomes from this clade (RBG-16-68-12 in GTDB) from the RBG dataset from Rifle, CO aquifer sediments [28]. Given that this clade is now represented by 35 species-level genomes (with 11 additional species added in this study), and that 12 genomes satisfy the completeness and contamination requirements to be considered high-quality drafts [35], we propose that they define a new candidate order, hereafter referred to as the Ca. Angelarchaeales. We note that our phylogenetic reconstruction also provides strong bootstrap support for nesting of a previously reported genome within the Ca. Angelarchaeales, Ca. Lunaplasmatales lacustris [36], proposed to represent an order-level lineage, the Ca. Lunaplasmatales (Supplementary Fig. 7). Given there are likely at least three families within Ca. Angelarchaeales, and as Ca. Lunaplasmatales lacustris is the only representative of one of these families, we propose the Ca. Lunaplasmatales be maintained as a family-level lineage within the Ca. Angelarchaeales order. Of the Ca. Angelarchaeales genome set, 20 contain identifiable *pmo*/*amo* gene clusters. The fact that CuMMO sequences are encoded within genomes of a single monophyletic subclade of the Thermoplasmatota is similar to previous observed patterns of CuMMO distribution, as taxa that encode CuMMO systems are often constrained to monophyletic groups scattered across the tree of life [3, 17, 19]. Despite likely incomplete sampling of the Ca. Angelarchaeales lineage, it appears that the presence of CuMMOs is not conserved across the order (similarly to the Nitrososphaerales). Some deeper branching genomes which likely have different family-level membership such as Angelarchaeales-34, Angelarchaeales-6, and the Ca. Lunaplasmatales lacustris genome metabolically characterized in a separate work [36] lack CuMMOs.

### Ca. Angelarcheales can occur at high relative abundance in some environments

Using ribosomal protein L6 (rpL6) as a taxonomic marker, we determined an average prokaryotic community relative abundance of 1.73 ± 2.25% for Ca. Angelarchaeales in 185 samples taken from six environments where the genomes of these organisms have been previously recovered (Fig. 2B and Supplementary Tables 5–7). In comparison, the average relative abundance of Nitrososphaerales in the same dataset was 0.65 ± 0.61%. We also assessed the relative abundance of *amoA*/*pmoA* as a functional marker and found that the frequency of *amoA*/*pmoA* reads associated with Ca. Angelarchaeales and Nitrososphaerales generally agreed with the relative abundance frequencies calculated using rpL6 (Supplementary Fig. 8).

### Ecological abundance associations of Ca. Angelarchaeales with other microbial taxa

Using the rpL6 abundance information we constructed a co-occurrence association network between all order-level taxa identified. We could reliably detect three sub-network modules and observed that while two modules formed separate large groups of interconnected taxa (modules 1 and 2), a third module (module 3), which contained the Ca. Angelarchaeales, appeared to bridge modules 1 and 2 (Supplementary Fig. 9a, b and Supplementary Tables 7, 8). We found the nodes representing order-level taxa in module 3 had significantly higher bridging centrality values on average when compared to nodes from each of the other two modules, indicating their tendency to exist between and connect modular network components (Supplementary Fig. 9c; FDR_3v1_ = 0.00016, FDR_3v2_ = 2e-5; Pairwise-Wilcoxon test). The Ca. Angelarchaeales have significant positive associations with 18 other order-level taxa (Supplementary Fig. 9d). The strongest association was with the unnamed order 40CM-2-53-6 (rho = 0.752, FDR < 0.001) within the Bathyarchaeia, a group of archaea widely distributed in soils and sediments with broad capacities for detrital peptide and carbohydrate degradation [37]. Ca. Angelarcheales also shared positive associations with Nitrososphaerales (Fig. 2B; rho = 0.366, FDR < 0.001) and Nitrospirales, nitrifiers which also were strongly associated with the Nitrososphaerales (rho_Ang_ = 0.400, FDR < 0.001; rho_Nitroso_ = 0.613; FDR < 0.001; Supplementary Fig. 9e–g).

### General metabolic features of Ca. Angelarchaeales

We conducted a general metabolic analysis of Ca. Angelarchaeales to place the encoded CuMMOs into metabolic context (Fig. 2C, Supplementary Fig. 10, Supplementary Table 9, and Supplementary Data 2). Ca. Angelarchaeales genomes contained many of the electron transfer and ammonia assimilation components known to be conserved in characterized AOA including: an NADH:ubiquinone oxidoreductase complex with an additional copy of the M protein component (CPLX 1), a four-subunit putative succinate dehydrogenase complex (CPLX 2), a complete cytochrome b containing complex III with a plastoquinone-like electron transfer apparatus (CPLX 3), up to two distinct oxygen reducing terminal oxidases (CPLX 4), an ammonia transporter (*amt*), a glutamine synthase (*glnA*), and glutamate dehydrogenase (*gdhA*). We found little evidence that Ca. Angelarchaeales can use inorganic nitrogen or sulfur containing compounds as alternative electron acceptors, thus it is likely that these organisms are obligate aerobes. We also did not identify carbon fixation pathways within any CuMMO encoding Ca. Angelarchaeales.

**Fig. 3:**
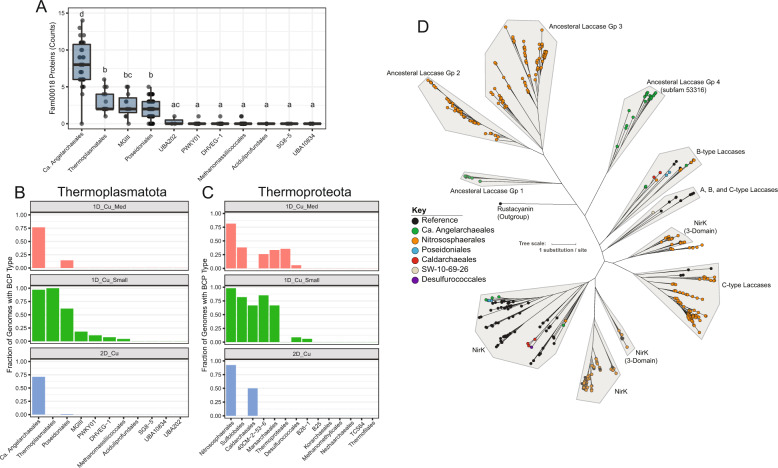
Analysis of BCPs across CuMMO encoding archaeal phyla. **A** Counts of fam00018 proteins in genomes from each order level lineage of the Thermoplasmatota containing ≥3 genomes. Each dot represents one genome. Boxes indicate the first and third quartile of counts, lines in boxes indicate median values, and whiskers indicate 1.5 × IQR in either direction. Letters above boxes indicate statistically significant differences between groups. Groups sharing no letters have statistically significant differences (FDR ≤ 0.05; pairwise-Wilcoxon test). **B** The fraction of genomes within each order level lineage of the Thermoplasmatota containing ≥ 3 genomes carrying the BCP subtype noted in the plot title. **C** The fraction of genomes within each order level lineage of the Thermoproteota containing ≥ 3 genomes carrying the BCP subtype noted in the plot title. **D** Maximum likelihood phylogenetic protein tree containing 349 2-domain and 3-domain BCPs from our analysis and 90 reference NirK and 2-domain laccase proteins. Clades were manually defined, shaded in gray, and named based on their constituent reference sequences or based on their sequence architecture relative to expected ancestral 2-domain BCPs. Node colors indicate if a sequence was a reference sequence (black) or the order level taxonomy of the encoding genome. Rustacyanin (ACK80662.1) is provided as an outgroup. Tree scale indicates branch distance for 1 mean substitution per site.

Some genomes encode a credible nitrite reductase (*nirK*) and 2-domain cupredoxins with homology to *nirK* (*nirK*-like). However, *nirK* is not essential for ammonia oxidation in Nitrososphaerales [20]. Also identified were plastocyanin-like proteins, which are common in Nitrososphaerales (Fig. 3), and three distinct copper transport systems (Fig. 2C). We did not detect any genes with homology to methanol dehydrogenases (*mdh*/*xoxF*) across the entire Ca. Angelarchaeales clade. However, several divergent secondary alcohol dehydrogenases were present in Ca. Angelarchaeales genomes. In addition, mechanisms to assimilate formaldehyde and formate (breakdown products of methanol) were present.

Ca. Angelarchaeales contain numerous transporters for branched chain amino acids, polar and non-polar amino acids, oligopeptides, and many proteases (Supplementary Fig. 10b–d). The number of encoded amino acid and peptide transport systems in Ca. Angelarchaeales is on average the largest across the phylum Thermoplasmatota (Supplementary Fig. 10c). The presence of a branched chain keto acid dehydrogenase complex (BCKDH) enables the degradation of branched chain amino acids to acetyl and propionyl-CoA, and a glyoxylate shunt (*aceA* and *aceB*) enables the carbons of acetyl-CoA to be used for biosynthesis. Sevral enzymes indicate the capacity for acetate degradation to acetyl-CoA (acetate-CoA ligase (*acdB*)) and lactate degradation to pyruvate (D-lactate dehydrogenase (*dld*)). These archaea do not have a complete glycolytic pathway (missing core enzymes including glucokinase (*glk*), phosphofructokinase (*pfk*), and pyruvate kinase (*pfk*)), but have gluconeogenesis pathways, thus enabling the biosynthesis of glucose from acetyl-CoA and pyruvate.

### Blue copper proteins (BCPs) are enriched in CuMMO containing archaea

As it has been previously postulated that BCPs may play important metabolic roles in CuMMO encoding archaea [17], we compared the BCP inventories in genomes of the phyla Thermoplasmatota and Thermoproteota, which include Ca. Angelarchaeales and Nitrososphaerales, respectively. The dataset included 34 representative Ca. Angelarchaeales genomes and 610 reference genomes (Supplementary Table 4). Due to their high primary sequence diversity, the identification and comparison of BCPs across organisms is difficult using standard annotation methods. Thus we clustered 1,103,913 proteins (Supplementary Data 2) using a previously validated two-step protein clustering approach [38]. This generated 76,216 protein subfamily clusters (subfams), which are groups of proteins sharing global homology, and 19,828 protein family clusters (fams), which are groups of protein subfamilies where remote local homology could be confidently detected. We identified 1927 proteins with BCP-associated (cupredoxin-like) PFAM domains across 30 protein fams (Supplementary Fig. 11a). Notably, a single protein family (fam00018) contained 1738 (90.2%) of these proteins, and the remaining proteins either made up very small fractions of other fams or were part of fams with very few proteins (Supplementary Fig. 11b). Analysis of the domain architectures of proteins within fam00018 indicate that this protein family primarily contains BCPs with between 1 and 3 cupredoxin-like domains. Included in fam00018 are small globular plastocyanin-like proteins, *nirK*-like proteins, two-domain laccase-like proteins, and the Cu binding cytochrome c oxidase subunit 2 (*coxB*/COX2) (Supplementary Fig. 11c). Fam00018 also contained 671 proteins with no identifiable domain annotations, which was expected given the high sequence diversity of BCPs. However, many proteins with no annotations were clustered into fam00018 subfamilies containing proteins with identifiable BCP domains, allowing the recruitment of these proteins into our analyses. We used the proteins of fam00018 as a broad homology group to quantify and ultimately sub-classify BCP types across genomes (Supplementary Table 10). Compared to all other order-level lineages in the Thermoplasmatota, Ca. Angelarchaeales genomes are significantly enriched in fam00018 proteins *(FDR* *<* *0.05; pairwise wilcoxon test)*, encoding on average 8.1 per genome (Fig. 3A). This pattern of fam00018 protein enrichment is similarly observed for the ammonia-oxidizing Nitrososphaerales order, which encode 13.3 per genome on average, relative to sibling orders within the Thermoproteota (FDR < 0.05; pairwise wilcoxon test; Supplementary Fig. 10d). It has recently been observed that some families within the Nitrosospherales order do not encode AMOs [39]. Here we found that the average number of BCPs per genome in Nitrososphaerales families that encode AMOs is statistically higher relative to those that do not (*p* value = 0.032; Wilcoxon test; Supplementary Fig. 11e). Nonetheless, this may still be a general feature of CuMMO encoding archaeal organisms rather than one related to substrate specificity.

### Subclassification of fam00018 identifies specific BCP architectures associated with lineages carrying CuMMOs

To more comprehensively understand the subtypes of BCPs that are present across the archaeal orders within Thermoplasmatota and Thermoproteota, we subdivided fam00018 into six manually annotated groups that covered 85.3% of all fam00018 proteins (Fig. 3B, C, Supplementary Fig. 12a, b, and Supplementary Table 10). We observed that small plastocyanin-like 1-domain BCPs (<250 aa), while present in many lineages, were extremely prevalent in the genomes of Ca. Angelarchaeales and Nitrososphaerales, supporting their important role in facilitating electron transport in these groups (Fig. 3B, C). Alternatively, medium length 1-domain BCPs (250-400 aa) and two-domain BCPs were encoded by most genomes of Nitrososphaerales and Ca. Angelarchaeales, found in few lineages outside them, and if found were not widely present in the genomes of those other lineages (Fig. 3B, C). This is consistent with these proteins performing functions that are specific to both Ca. Angelarchaeales and Nitrososphaerales.

The two-domain cupredoxins (two-domain BCPs which include *nirK*), can be differentiated based on phylogenetic relationships, the types of copper centers they contain, and the arrangement of these centers [40, 41]. A phylogenetic tree for two-domain BCPs, known *nirK* sequences (which include three-domain BCPs), and two-domain laccase sequences (Fig. 3D) resolves 11 discrete clades. The Nitrososphaerales *nirK* clades are distinct from classic *nirK* sequences, as has been observed previously [42]. The four high confidence *nirK* sequences identified in Ca. Angelarchaeales fall into the classic *nirK* clade. Four clades are composed of sequences that contain two Type I copper centers but appear to lack Type II or III centers. Such proteins lack functional predictions, and are referred to as ancestral forms of two-domain BCPs [40]. The two-domain BCPs of ancestral group 4 (subfam53316) and ancestral group 1 (subfam54500) are exclusively found in Ca. Angelarchaeales. We also note that while two-domain BCPs were found in archaeal orders outside of Ca. Angelarchaeales and Nitrososphaerales, these sequences fall into clades of known laccases.

### BCPs in Ca. Angelarcheales are co-localized with energy conversion machinery

**Fig. 4:**
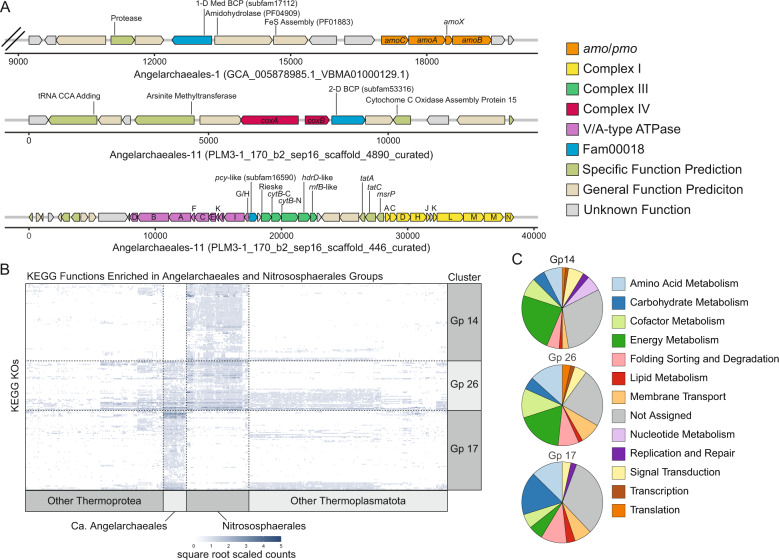
Genomic context and metabolic function enrichment analysis for Angelarchaeales. **A** Example operons showing the gene context surrounding the amo/pmoCAXB gene cluster (top), the coxAB oxygen utilizing terminal oxidase gene cluster (middle), and the complex III like cytochrome b gene cluster (bottom). Subfam membership is noted in labels for fam00018 proteins (blue). Scale is in base pairs and double hash on amo/pmoCAXB containing contig indicates truncation after ten genes to the left of the cluster for readability. **B** Heatmap showing the number of hits to 201 KEGG KOs found to be significantly enriched in Angelarcheales (Gp 17), the Nitrososphaerales (Gp 14), or showing shared enrichment by both orders relative to all others (Gp 26). Each column represents the hits across one genome, and each row represents the hits for a single KO. Intensity of each spot in the heatmap is based on square root scaled counts of hits to a KO in each genome for ease of readability. Dotted lines are added to segregate clusters for ease of viewing. **C** Breakdowns of functional categories associated with KEGG KOs in each enrichment group. Also see Supplementary Table 14.

We examined the genomic context, in Ca. Angelarchaeales, of three gene clusters known to be important for electron transfer and energy generation in CuMMO encoding archaea: the *amo*/*pmoCAXB* cluster, the *coxAB* oxygen utilizing terminal oxidase cluster, and the complex III like cytochrome b gene cluster (Fig. 4A and Supplementary Figs. 2, 13, and 14). Four of the *amo*/*pmoCAXB* encoding contigs from 20 genomes encode a medium length 1-domain BCP from subfam17112 ~4 genes upstream of the *amo*/*pmoCAXB* gene cluster. We note that only 5 of 20 contigs have sufficient length upstream of the *amo*/*pmoCAXB* locus to allow identification of this BCP. The proteins of subfam17112 are all predicted to contain 5 transmembrane helices in their N-terminal region with a cupredoxin-like domain occupying the outer membrane facing C-terminal region (Supplementary Fig. 15). The eight proteins of subfam17112 only occur in Ca. Angelarcheales genomes that also encode an *amo*/*pmo*. Medium length 1-domain BCPs also occur at a high frequency in Nitrososphaerales (Fig. 3C) and have been previously proposed as putative candidates for the missing hao activity [16]. Thus, while the enzymatic activity of subfam17112 is as yet undetermined, its proximity to the *amo*/*pmoCAXB* locus is intriguing.

We reconstructed *coxAB* encoding contigs from 29 genomes. In 19 of these genomes we could identify a two-domain BCP directly following the *coxB* gene, which in 14 of 19 cases was the two-domain BCP from the *nirK*-like group subfam53316 (Fig. 3D). Again, in at least seven cases it was not possible to search for a BCP as contigs were of insufficient length.

A cytochrome b containing complex III-like locus could be identified and reconstructed in 30 genomes. It was commonly co-located with gene clusters encoding other components of the electron transport chain (the V/A-type ATPase and NADPH:Quinone oxidoreductase - complex I). Electron transfer from complex III to downstream electron transport machinery is posited to involve a plastocyanin-like 1 domain BCP, not a soluble cytochrome c, similar to ammonia oxidizing Nitrososphaerales [16, 43]. In Ca. Angelarchaeales, we found a small 1 domain plastocyanin-like BCP gene upstream of a Rieske iron-sulfur protein in 23 of 30 reconstructed complex III loci (and no cytochrome c).

### Metabolic functions enriched in Ca. Angelarchaeales and Nitrososphaerales

Using indicator analysis, we identified KEGG orthology groups (KOs) that were significantly enriched in the Ca. Angelarchaeales (Gp 17) individually, the Nitrososphaerales (Gp 14) individually, and the KOs shared by both orders relative to all other orders of Thermoplasmatota and Thermoproteota (Gp 26). Of the 78 KOs that were significantly enriched in Ca. Angelarchaeales (Fig. 4B and Supplementary Table 11), the largest functional groups corresponded to carbohydrate metabolism (17.2%), amino acid metabolism (12.6%), and protein folding, sorting, and degradation (10.3%) (Fig. 4C). KOs enriched in Ca. Angelarchaeales support the use of peptides and amino acids as a carbon and nitrogen source. These included isocitrate lyase of the glyoxylate shunt (K01637), proteins for detoxification of the threonine catabolite methylglyoxal (K10759, K18930, and K23257), 4 proteases (K01392, K06013, K07263, and K09640), components of the archaeal proteosome (K13527 and K13571) enzymes for betaine (K00130, K00544, and K00479), proline (K00318), and cysteine (K01760) catabolism, the E1 component of the branched chain keto acid dehydrogenase complex (K00166), and a transport system for polar amino acids (K02028 and K02029).

The 75 KOs significantly enriched in Nitrososphaerales genomes were largely associated with energy metabolism (23.7%) (Fig. 4C). This included functions critical for the hydroxypropionate/hydroxybutyrate carbon fixation pathway known to operate in these organisms (K18593, K18594, K18603, and K18604). Other functions enriched in Nitrososphaerales are involved in electron transfer including plastocyanins (K02638), ferredoxins (K05524), and rieske iron-sulfur proteins (K15878). We also identified enriched capacity for urea utilization (K01429, K01430, K03187, K03188, K03190) and urea transport (K20989), which agrees with the fact that many Nitrososphaerales are thought to use urea as a nitrogen source for ammonia oxidation [44].

The amo/pmo subunits A and C (K10944 and K10946) were identified among the 48 functions that were significantly enriched in both Ca. Angelarchaeales and Nitrososphaerales compared to the other groups. Many shared functions were also associated with energy metabolism and have been identified previously as important metabolic features in CuMMO encoding Nitrososphaerales, including nitrite reductase (K00368), the oxygen utilizing terminal oxidase subunit I (K02274), the ammonium transporter (K03320), a duplicated NADPH:Quinone oxidoreductase subunit M (K00342), and a split cytochrome b-561 like protein (K15879), as well as three iron-sulfur complex assembly proteins (K09014, K09015, and K13628), a cytochrome c oxidase complex assembly protein (K02259), a high affinity iron transporter (K07243), and a copper transporter (K14166).

### Metabolic reconstruction supports the feasibility of an amino acid based metabolism

**Fig. 5:**
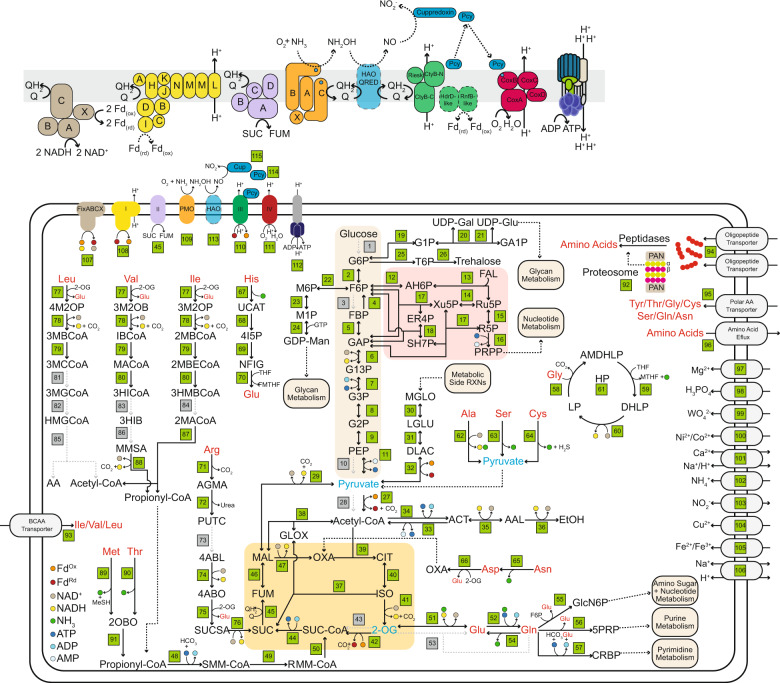
Metabolic reconstruction of Angelarchaeales-1 genome. For full reaction, gene list, and list of compound abbreviations see Supplementary Tables 12–14. Green boxes indicate a reaction (and its reference number in Supplementary Table 13) that could be linked to a gene with the predicted metabolic function. Black arrows with solid lines indicate a reaction that could be identified, associated smaller arrows with colored dots indicate consumed, and generated reaction substrates and products are indicated in the key at the bottom left. Black arrows with dotted lines indicate flow of metabolites to other pathways or reactions. Gray arrows with dotted lines and gray boxes indicate reactions that were searched for and could not be identified. Amino acids are in red text to highlight their locations throughout the figure. Metabolites in blue text indicate hubs for carbon derived from amino acid catabolism. For ease of viewing the reactions of glycolysis, the pentose phosphate pathway, and the TCA cycle have been highlighted with beige, red, and orange backgrounds. The upper panel is a blow up of the electron transport reactions showing predicted organizations of subunits in each complex. Reference numbers for each subunit can be found in the larger figure panel, and colors of subunits are the same as those used in that figure panel. Transparent HAO-QRED indicates a putative/proposed functionality. Black arrows with dotted lines indicate putative reactions. Protein subunits with solid color but dotted borders indicate a protein was found but functionality is unclear.

We undertook a complete metabolic reconstruction on the Angelarchaeales-1 genome to evaluate the feasibility of a life strategy where amino acid metabolism could be coupled to ammonia oxidation. We focused on pathways for the import and catabolism of amino acids and routes by which their products feed into central carbon metabolism, ammonia oxidation, and are interconnected with electron transport and energy generation (Fig. 5 and Supplementary Tables 12–14).

The Angelarchaeales-1 genome lacks key oxidative enzymes of glycolysis and oxidative enzymes of the pentose phosphate pathway. It can interconvert glucose/fructose to mannose and galactose derivatives but appears unable to import or phosphorylate these sugars. The encoded fructose bisphosphatase would allow gluconeogenesis. It has no detectable pyruvate kinase and instead encodes a pyruvate orthophosphate dikinase, which is known to be allosterically regulated and reversible in archaea [45, 46]. The capacity for production of the compatible solute trehalose is notable, as Angelarchaeales-1 derives from an environment that regularly undergoes large cyclic changes in water content [25].

Angelarchaeales-1 encodes a full complement of genes for the conversion of pyruvate into Acetyl-CoA, the TCA cycle, and a glyoxylate shunt. However, it lacks genes for both the pyruvate dehydrogenase complex as well as the 2-oxoglutarate dehydrogenase complex. These reactions are likely enabled by pyruvate/2-oxoglutarate ferredoxin oxidoreductase systems, which provide reduced ferredoxin. A glyoxylate shunt allows for catabolic reactions that terminate in 2-carbon compounds (e.g., acetyl-CoA produced by amino acid and acetate catabolism) to be utilized for biosynthetic purposes, as it bypasses the two decarboxylation steps of the TCA cycle.

We identified reasonably confident catabolic routes for 15 amino acids, including a complete glycine cleavage system, as well as a route for the end product (propionyl-CoA) of at least four amino acid catabolic pathways, to be incorporated into the TCA cycle as succinyl-CoA. Genes encoding the terminal reactions of branched chain amino acid degradation were not identified, although the genome encodes numerous acyl-CoA dehydrogenases with unknown specificity that could perform these functions. However, we could confidently identify the branched chain keto-acid dehydrogenase complex (BCKAD) that is critical for the degradation of leucine, isoleucine, and valine, as well as in processing the downstream degradation products of methionine and threonine. Finally, this organism carries multiple independent branched chain amino acid transport systems, as well as a polar amino acid transport system that is enriched in the Ca. Angelarchaeales order (Fig. 4B, Supplementary Fig. 10c, d, and Supplementary Table 13).

Angelarchaeales-1 and ammonia oxidizing Nitrososphaerales both have respiratory chains that include a complex I lacking the E, F, and G subunits for NADH binding and a duplicated subunit M that may mediate translocation of an additional proton [47]. The electron donor to the complex I may be reduced ferredoxin [47, 48]. Both groups encode a four-subunit succinate/fumarate dehydrogenase, a cytochrome b-like complex III with an associated plastocyanin-like electron transfer protein, and an oxygen utilizing cytochrome c terminal oxidase complex.

Unlike the Nitrososphaerales, Angelarchaeales-1 encodes a multitude of systems for the putative utilization of ferredoxin. This includes a FixABCX electron bifurcation system that can couple the reduction of ferredoxin and quinone to the oxidation of NADH. Interestingly the FixABCX complex is co-located with the BCKAD complex in the Angelarchaeales-1 genome. This FixABCX complex may be important for converting the reducing power of NADH derived from BCKAD mediated branched chain amino acid degradation into reducing power in the form of reduced ferredoxin and quinone. Angelarchaeales-1 has 2 unusual genes proximal to the cytochrome b-like complex III, a *hdrD*-like gene and a *rnfB*-like gene. In rnf complexes, *rnfB* binds and oxidizes reduced ferredoxin. Thus, complex III may act as another entry point for reduced ferredoxin into the respiratory chain.

## Discussion

In this study, we identified a novel CuMMO group occurring in a largely unstudied lineage of archaea, the Ca. Angelarchaeales, that can exist at potentially high abundance in some soils, terrestrial sediments, and are even present in some deep ocean systems. Several lines of informatic evidence presented here support these novel proteins as genuine and catalytically active members of the CuMMO group including: (i) Significant global homology and high-quality alignment with known reference CuMMO sequences displaying conservation of key residues required for CuMMO activity [31], and (ii) a predicted structure of a novel CuMMO catalytic subunit (*amoB*/*pmoB*) with a similar fold and shared structural superposition of key functional residues with a known *amoB* protein from Nitrosocaldus yellowstonii (PDB structure 4O65_A). However, this new CuMMO group is phylogenetically very distant from any known CuMMO proteins, forming a third major group, greatly expanding their known diversity. While future work will be required to confirm the exact substrate specificities for this new class of enzymes, we explore here a feasible hypothetical scenario for ammonia oxidase functionality considering the metabolic and ecological context of the archaea where these enzymes are encoded. The conclusive identification of ammonia, methane, or hydrocarbons as the substrate for these enzymes would mark the first, first, or second known lineage of archaea with the ability to perform aerobic methane, hydrocarbon, or ammonia oxidation respectively. Regardless, the identification of a novel group of CuMMO enzymes in an archaeal lineage outside of the Nitrososphaerales has important implications for the evolution and distribution of these enzymes within archaea and suggests that there are overlooked archaeal groups contributing to critical aerobic nitrogen and/or carbon biogeochemical cycling functions.

The Ca. Angelarchaeales, named herein, were a largely understudied lineage within the Thermoplasmatota. While our analysis of their abundance was limited to environments where their genomes have been previously detected, and we acknowledge that these data cannot represent all soils and sediments, it is striking that Ca. Angelarchaeales can comprise a reasonably large fraction of some communities in which they were detected. In addition, it is likely that the inferred relative abundance of these organisms reflects species that also in fact encode CuMMOs as our *amoA*/*pmoA* marker-based analysis closely mirrors abundance distributions inferred from the phylogenetic marker rpL6. It is also intriguing that in the environments studied these organisms occupy a co-occurrence cluster that bridges more closely interconnected groups of microbial taxa. Thus, Ca. Angelarchaeales may provide an intermediary or high-level biogeocheomical cycling function which is consistent with roles in the turnover of proteinaceous detritus or the conversion of methane and/or ammonia into oxidized forms to be used by other organisms.

Metabolically we find that Ca. Angelarchaeales are likely obligate aerobes and carry extensive capabilities for amino acid and peptide uptake and catabolism. While we present a possible scenario where ammonia liberated from peptide and amino acid degradation could be aerobically oxidized by the novel CuMMOs, the proposed metabolism does not preclude the feasibility of methane or both ammonia and methane as being possible CuMMO substrates. Indeed the Ca. Angelarchaeales have the capability to incorporate formaldehyde into central carbon metabolism via the RuMP pathway and their genomes encode numerous divergent secondary alcohol dehydrogenases that may have the capability to oxidize methanol.

The large repertoire of BCPs in Ca. Angelarchaeales genomes relative to sibling orders of the Thermoplasmatota is of particular interest, as previous work and our evidence here suggest that large repertoires of BCPs may be an important feature of CuMMO encoding archaeal groups. In ammonia oxidizing Nitrososphaerales it has been postulated that BCPs could perform the yet unresolved enzymatic steps that oxidize hydroxylamine to nitrite [16, 17]. However, the specific BCPs present in any Nitrososphaerales genome are highly variable [16], and in Ca. Angelarchaeales we observe a similar pattern, i.e., an extensive inventory of BCPs relative to sibling lineages with no individual BCP ortholog being conserved across the entire group. Generally, we do observe that sets of 2-domain BCPs and 1-domain medium length BCPs with divergent sequences but conserved architecture are largely restricted to the Ca. Angelarchaeales and Nitrososphaerales lineages. In addition, we observe a combination of electron transport proteins that is shared by Ca. Angerlarchaeales and Nitrosospherales, consistent with similar electron transport chain functionality and energy conversion strategies.

Some ecological support for the hypothesis that Ca. Angelarchaeales are ammonia oxidizers is their strong association with, and high abundances in, relatively aerobic grassland soils, where organisms with the capability for methanogenesis have not been detected [25, 49]. These archaea also occur in sediments from above the water table and in saturated sediments that periodically receive oxygenated water [50]. In this context, it is interesting to note that the Ca. Angelarchaeales can utilize amino acids as a carbon and nitrogen source without oxygen, generating energy via substrate level phosphorylation (as we detect the ability to ferment acetyl-CoA to ethanol), and oxidizing ammonia when O_2_ is available.

Overall, the identification of a lineage of archaea outside of the Nitrososphaerales encoding CuMMOs has important implications for understanding how these enzymes and microbial groups contribute to carbon and nitrogen biogeochemical cycling. Ca. Angelarchaeales likely occur in a variety of soils and sediments where they have so far gone undetected using primer based CuMMO survey methods. Thus, this new clade of CuMMOs provides an opportunity for further study of these enzymes, and for molecular clock-based analyses of their evolution relative to Earth history [51].

## Methods

### Amo/Pmo identification, genome set selection, and de-replication

Previous genome-resolved work at the Angelo Coast Range Reserve [25] identified seven Thermoplasmatota genomes each containing a hit to the KOFAM HMM for *amoA*/*pmoA* (K10944), below the HMM score threshold, but with a significant E-value (≤1E^−4^). Proteins co-localized with these divergent *amoA*/*pmoA* proteins were identified. To search for additional similar divergent *amoA*/*pmoA* proteins we aligned the divergent proteins using MAFFT v7.471 (--maxiterate 1000 --localpair) and constructed an HMM using hmmbuild in the HMMER v3.3.1 package [52] with default parameters. This HMM was scored against all archaeal genomes in the GTDB release r95 [53], and all archaeal MAGs in ggKbase (ggKbase.berkeley.edu) datasets as of January 13, 2020 using the hmmsearch function of the HMMER package with an HMM score threshold of 100. These genomes were phylogenetically classified using the GTDB tool kit [54] (GTDB-tk) classify workflow with default parameters. All genomes were placed within the RBG-16-68-12 order (hereafter Ca. Angelarchaeales) of the GTDB taxonomy (Supplementary Table 1).

To produce a full genome reference set for our analyses we added an additional 31 genomes that did not have any hit to our custom *amoA*/*pmoA* HMM but fell within the Ca. Angelarchaeales order (Supplementary Table 1). These genomes came both from Angelo Coast Range Reserve assemblies (*n* = 15 genomes) and from the Ca. Angelarchaeales order in GTDB (*n* = 16 genomes). We also added 719 reference genomes from GTDB derived from the archaeal phyla Thermoplasmatota (*n* = 338 genomes) and Thermoproteota (*n* = 371). Thermoproteota genomes were included for comparison of functional differences within and between phyla of known (Nitrosospherales) and putative (Ca. Angelarchaeales) AOA.

The full genomes set was de-replicated at the species level (Average Nucleotide Identity ≥ 95%) using dRep [55] with the following parameters: -p 16 -comp 10 -ms 10000 -sa 0.95. The best genome from each species cluster was chosen as a representative genome by dRep. Species representatives were required to have ≥60% completeness and ≤10% contamination as estimated by checkM [56]. If no genome within a species cluster met these criteria the cluster was discarded. All genome information can be found in Supplementary Tables 2 and 4.

### Amo/Pmo protein identification, alignment, analysis, and phylogenetic reconstruction

The *amoA*/*pmoA* protein was used as an anchor sequence to manually annotate putative *amoB*/*pmoB*, *amoC*/*pmoC*, and *pmoX* proteins present on each contig across the Ca. Angelarchaeales genomes (Supplementary Table 2). Manual annotation used a combination of gene order relative to the *amoA*/*pmoA* sequence, predicted protein length (compared to known amo/pmo proteins), and best blast hits vs. the NCBI nr database.

To identify *amoB*/*pmoB* and *amoC*/*pmoC* in Ca. Angelarchaeales genomes where no *amoA*/*pmoA* was identified, and to develop a method to rapidly identify all novel *amo*/*pmo* complex proteins in future work we aligned each set of *amo*/*pmo* proteins following manual annotation using MAFFT v7.471 (--maxiterate 1000 --localpair) and constructed HMMs for each using hmmbuild in the HMMER v3.3.1 package with default parameters. These were scored against all proteins in the full set of 228 redundant Ca. Angelarchaeales genomes (Supplementary Table 1) using the hmmsearch function of the HMMER v3.3.1 package with an HMM score threshold of 100.

Alignments of putative *amo*/*pmo* sequences with known references were constructed by merging amo/pmo sequences from Ca. Angelarchaeales were with reference sequences from Khadka, et al. [1] and those from an additional set of 22 ammonia oxidizing archaeal reference genomes (Supplementary Table 3). This resulted in protein sets for *amoA*/*pmoA*, *amoB*/*pmoB*, and *amoC*/*pmoC* that contained 112, 114, and 110 sequences respectively. Sequence sets were aligned using MAFFT v7.471 with the following parameters: --maxiterate 1000 --localpair --reorder --thread 12. Mean amino acid identity between Ca. Angelarchaeales, bacterial, and archaeal reference sequences were calculated in Geneious Prime v2020.2.4 from a pairwise sequence identity matrix. Conserved residues for methane and AMOs were referenced from Wang, et al. [31] and identified in each alignment through manual inspection. An assessment of alignment column confidence was performed for the *amoB*/*pmoB* and *amoC*/*pmoC* alignments using the Guidance2 web server with default parameters (http://guidance.tau.ac.il/) [57].

A de novo predicted structure was inferred for the *amoB*/*pmoB* protein from the Angelarchaeales-1 genome using AlphaFold v2.0 implemented in the AlphaFold Google Colab iPython notebook with default parameter settings [33]. The predicted protein structure was searched for a best hit against the PDB database using the Vector Alignment Search Tool with default parameters [58]. Structural superposition and alignment between the predicted *amoB*/*pmoB* structure and the PDB structure 4O65_A was performed using the PDB structural alignment web server (https://www.rcsb.org/alignment) with the jFATCAT flexible algorithm with default parameters [59].

Maximum likelihood phylogenetic trees were constructed for each individual *amo*/*pmo* subunit alignment using IQ-TREE v1.6.12 [60] with the following options: -m MFP -bb 1000 -alrt 1000 -nt 12. The empirically selected evolutionary rate model for all *amo*/*pmo* sequence sets was LG + F + I + G4 based on Bayesian information criteria (BIC). Branch support was estimated using ultrafast bootstrapping with 1000 bootstrap replicates. Individual *amo*/*pmo* protein trees can be found in Supplementary Fig. 6. For the combined *amoABC*/*pmoABC* tree, sequences from the same organism were concatenated in Geneious Prime v2020.2.4 and only retained if at least 2 of the proteins were present, resulting in 112 total concatenated sequences in the alignment. A Maximum likelihood phylogenetic tree was constructed for concatenated sequences using IQ-TREE v1.6.12 with the following options: -m MFP -bb 1000 -alrt 1000 -nt 12. The empirically selected evolutionary rate model for the concatenated *amoABC*/*pmoABC* tree was LG + F + I + G4 based on BIC. Branch support was estimated using ultrafast bootstrapping with 1000 bootstrap replicates.

### Assessment of *amoA*/*pmoA* primer complementarity

Primer sequences used to amplify and quantify both archaeal (GenAOAF: 5′-ATA GAG CCT CAA GTA GGA AAG TTC TA-3′; GenAOAR: 5′-CCA AGC GGC CAT CCA GCT GTA TGT CC-3′) and bacterial (amoA-1Fmod: 5′-CTG GGG TTT CTA CTG GTG GTC-3′; GenAOBR: 5′-GCA GTG ATC ATC CAG TTG CG-3′) *amoA*/*pmoA* sequences using PCR from environmental samples were obtained from Meinhardt, et al. [30]. Sequence complementarity was assessed using the map primers function of Geneious Prime v2020.2.4. Primers were tested against nucleotide sequences of *amoA* genes from Nitrososphaerales genomes and *amoA*/*pmoA* genes from Ca. Angelarchaeales genomes as detailed in Supplementary Table 3. A match required both the forward and reverse primers to bind to the sequence while allowing up to 7 mismatched bases in each primer. Full data obtained for the number of mismatches and estimated product sizes for each primer pair and template sequence are available in Supplementary Table 3.

### Genome taxonomy and phylogenetic reconstruction

Initial taxonomic placement for the 645 non-redundant genomes used in this study was performed using the GTDB-tk [54] classify workflow with the following parameters: classify_wf -x fasta --cpus 48. All GTDB-tk based taxonomic classification is available in Supplementary Table 4. A concatenated marker gene phylogenetic tree for all genomes classified within the archaeal phylum Thermoplasmatota was constructed by combining the 34 Ca. Angelarchaeales genomes, the Ca. Lunaplasmatales lacustris genome (GCA_017885655.1), and 302 de-replicated reference genomes from Thermoplasmatota spanning all known orders. The *Archaeoglobus fulgidus* (GCF_000008665.1) genome was also included to be used as an outgroup for tree rooting. GToTree v1.5.22 [61] was used to identify and extract a set of 122 phylogenetically informative single copy archaeal marker genes (SCGs), defined in Rinke, et al. [34], from each genome using the following parameters: -H Archaea.hmm -j 8 -d. Genomes where <50% (61 genes) of the targeted marker genes could not be identified were removed from the analysis (*n* = 24 genomes) retaining a total of 313 genomes in the final tree. SCG sequence sets were then individually aligned with Muscle v3.8 [62] and alignments were trimmed with Trimal v1.4 [63]. All alignments were then concatenated, and an SCG alignment partition table was produced by GToTree so evolutionary substitution rate models could be estimated for each SCG independently during phylogenetic tree construction. A maximum likelihood phylogenetic tree was constructed with IQ-TREE v1.6.12 [60] with the following options: -spp Partitions.txt -m MFP -bb 1000 -alrt 1000 -nt 48. Evolutionary rate models were empirically estimated for each marker gene independently and selected based on BIC. Branch support was estimated using ultrafast bootstrapping with 1000 bootstrap replicates. Phylogenetic trees were rooted using *Archaeoglobus fulgidus* as an outgroup, annotated, and displayed using iTOL v6. For the full tree see Supplementary Fig. 7.

### RpL6 and *amoA*/*pmoA* marker abundance and association analysis

There are six study sites where at least 1 Ca. Angelarchaeales genome was reconstructed from shotgun metagenome data. We used all 185 shotgun metagenomic samples collected from these sites, regardless of whether a Ca. Angelarchaeales genome was recovered from a sample, to estimate the relative abundance of the Ca. Angelarcheales order across these locations (Supplementary Table 5). Relative abundance of all bacterial and archeal order level taxonomic groups was quantified using marker gene taxonomic placement and quantification of the phylogenetically informative SCG ribosomal protein L6 (rpL6). RpL6 marker gene profiling and quantification was performed using GraftM v0.13.1 [64]. Briefly, an rpL6 graftM database was constructed using the ribosomal L6 protein sequences from all archaeal and bacterial genomes (provided by GTDB using TIGR03653 and TIGR03654, respectively) in GTDB v95. A GraftM package was then created using this set of sequences and the rpL6 Pfam HMM (PF00347.24) sensitive for both bacterial and archaeal variants using the command: graftM create --sequences L6.faa --taxonomy taxonomy.tsv --hmm PF00347.hmm. GraftM was then used to call genes, identify rpL6 sequences, phylogenetically place sequences, and quantify read counts of rpL6 sequences identified in each sample using the following options: graftM graft --forward [forward reads] --reverse [reverse reads] --graftm_package rpL6_gpkg --threads 48. Raw counts in each sample were aggregated to the taxonomic rank of order using a custom R script (https://github.com/SDmetagenomics/AMO_Archaea_2021). Counts of rpL6 sequences that could only be resolved to taxonomic ranks higher than order level were retained in their original form. All aggregated count data is available in Supplementary Table 6. Relative abundances of the Ca. Angelarchaeales and Nitrososphaerales orders in each sample were calculated as the fraction of total reads in a sample that were associated with these order ranks. Plotting was performed in R using the ggplot2 package [65].

*AmoA*/*pmoA* marker gene profiling and quantification was performed using GraftM v0.13.1 [64]. Briefly, an *amoA*/*pmoA* graftM database was constructed using the *amoA*/*pmoA* protein reference sequences in Supplementary Table 3 and Supplementary Data 1. A custom taxonomy table was built for the sequences and assigned based on the taxonomy of the encoding genome. A GraftM package was then created using this set of sequences and taxonomy table with the command: graftM create –sequences amoA_pmoA.faa --taxonomy taxonomy.tsv. GraftM was then used to call genes, identify *amoA*/*pmoA* sequences, phylogenetically place sequences and quantify read counts of *amoA*/*pmoA* sequences identified in each sample using the following options: graftM graft --forward [forward reads] --reverse [reverse reads] --graftm_package rpL6_gpkg --threads 48. Relative abundances of the Ca. Angelarchaeales and Nitrososphaerales associated *amoA*/*pmoA* reads were calculated as the fraction of total reads in a sample that were associated with these order ranks. Plotting was performed in R using the ggplot2 package [65].

Association analysis between the abundances of each taxonomic group across samples (Supplementary Table 7) was conducted using the propr package [66] in R. Proportionality was chosen as an association measure over correlation as it is better suited for compositional data such as sequencing read counts [67]. Briefly, taxonomic groups were filtered such that only those with >5 counts in at least 33% of samples (*n* = 61 samples) were retained. This filtering was performed to remove taxonomic groups with extremely low counts from association comparisons as to avoid spurious associations. Following filtering 90.5% of the original count data was retained in each sample on average. Subsequently the rho proportionality metric was calculated for centered log-ratio normalized counts between all pairs of taxonomic groups using the following function: propr(count_matrix, metric = “rho”, p = 1000). The magnitude of proportionality that represented a significant association (FDR < 0.001) was calculated through a permutation based procedure implemented using the updateCutoffs function within the propr package as follows: updateCutoffs(L6_rho, cutoff = seq(from = 0.05, to = 0.40,by = 0.01), ncores = 12). Briefly, this method determines the significance for a range of given proportionalities through a non-parametric permutation-based method that calculates false discovery rate (FDR). It was determined that all rho values ≥0.25 were statistically significant with a FDR < 0.001. In addition, only positive rho association values were considered in the final analysis, as negative associations in compositional data are considered less reliable, even after compositional correction measures are applied [66]. All significant proportionality values are available in Supplementary Table 7. Co-occurrence association networks were constructed, visualized, and statistically evaluated using Gephi v0.9.2. Networks were displayed using the MultiGravity Force Atlas 2 layout with LinLog mode enabled. Node bridging centrality and sub-network module membership were calculated in Gephi v0.9.2 using default parameters. Statistical comparisons were performed in R and plotting was performed in R using the ggplot2 package [65]. The abundance values in the plotted pairwise comparisons between Ca. Angelarchaeales, Nitrososphaerales, and Nitrospirales use the centered log-ratio of counts in each sample as calculated by the propr package.

### Genome annotation

Reference genomes used in this study were queried using GTDB assigned taxonomic labels from the GTDB database v95. Information describing the original samples, assembly, and binning for all references can be found through their GI number in the NCBI assembly database, which is included along with genome size, completeness, and contamination information in Supplementary Table 4. For the 645 genomes passing completeness and contamination quality criteria, annotation was performed as follows. Genes and protein sequences were predicted using prodigal v2.3.6 [68] using the following options: prodigal -i [genome contigs] -a [proteins sequences out] -m -p meta. KEGG KO annotations were predicted using KofamScan [69] using HMM models from release r02_18_2020 with the following options: exec_annotation -p [hmm profiles] -k [hmm cutoffs] --cpu 48 --tmp-dir [temp dir] -o [output folder] [protein file]. As multiple KEGG HMMs can match to the same protein with scores exceeding their score cutoff thresholds, the HMM with the lowest *E*-value had its annotation transferred to the protein. If a protein did not match any KEGG HMM above the HMM cutoff threshold then the lowest *E*-value annotation was transferred. An *E*-value cutoff of <1e−10 was applied, above which no annotations were transferred, and genes were not assigned to a KO. Archaeal COG (arCOGs) annotations were predicted for all proteins using HMMs from EggNOG v5 [70] using the following options: hmmsearch --tblout [arCOG hit table] -E 0.0001 --cpu 10 All_arCOG.hmm All_Proteins.faa. We searched for *xoxF*/*mxaF*-like pqq-binding methanol dehydrogenases in genomes using the hmmsearch function of the HMMER package and a custom HMM from Anantharaman, K. et al. [28] with an HMM score threshold cutoff of ≥166 using the following options: hmmsearch --tblout [xoxF_mxaF hits] -T 166 --cpu 12 methanol_dehydrogenase_pqq_xoxF_mxaF.hmm All_Proteins.faa. Protease counts in each genome were determined via the METABOLIC pipeline (https://github.com/AnantharamanLab/METABOLIC) implemented using the following options: perl METABOLIC-G.pl -t 48 -m-cutoff 0.75 -in [input protein files] -kofam-db full -o [output annotations]. Custom R code for parsing and plotting METABOLIC outputs used for peptidase quantification can be found in our github repository. Pfam domain annotations were predicted by searching all proteins against the PfamA database release r32 [71] using the hmmsearch function of the HMMER package with the following options: hmmsearch --domtblout [pfam domain table] --cut_ga --cpu 10 Pfam-A.hmm All_Proteins.faa. Overlapping pfam domain matches to the same protein were resolved, and domain boundaries were established, using the cath-resolve-hits function of the cath-tools package with default parameters (https://github.com/UCLOrengoGroup/cath-tools). All annotations were aggregated into a final table using a custom R script which is available in our github repository. Also the complete gene level annotation table for all 645 genomes is available at FigShare (https://figshare.com/projects/AMO_Archaea/112599).

For functional annotations of genes and pathways across all Ca. Angelarchaeales genomes, a subset of 190 specific target functions were analyzed (Fig. 2b and Supplementary Fig. 10a). Pathways and protein complexes were subsequently grouped into 77 “gene groups” that represent the capability to perform a pathway or metabolic function. Each gene group had specific criteria that were required (i.e., a critical protein needed to be present) for a positive detection. All targets, gene group organization, and criteria for detection are available in Supplementary Table 9. For annotation and quantification of amino acid and peptide transport systems across all 645 genomes in our analysis a set of 38 KEGG KOs representing individual transporter proteins or transporter subunits were used as the search criteria (Supplementary Fig. 10c, d and Supplementary Table 15). For functional annotation of the Angelarchaales-1 genome and metabolic map reconstruction (Fig. 5), a subset of 233 specific target functions were analyzed that highlighted central carbon metabolism, the degradation of amino acids, and electron transport/energy generation. Pathways and protein complexes were subsequently grouped into 115 “reaction groups” that represent the capability to perform a pathway or metabolic function. Each reaction group had specific criteria that were required (i.e., a critical protein needed to be present) for a positive detection. All targets, reaction group organization, genes associated with reactions, and criteria for detection are available in Supplementary Table 13.

### Protein clustering

Clustering of all proteins predicted in the 645 genomes passing completeness and contamination quality criteria was accomplished as described in Méheust, et al. [38]. Code for this pipeline is available at: https://github.com/raphael-upmc/proteinClusteringPipeline. Briefly, all 1,103,913 proteins were first clustered into subfamilies (subfams) using the subfams.py script of the pipeline (which implements mmseqs2 [72] clustering) using the following options: subfamilies.py --output-directory [clustering dir] --cpu 48 --coverage 0.8 All_Proteins.faa. All proteins within a subfam must align with bidirectional coverage of at least 80% (--cov-mode 0 in mmseqs2), and alignments must have an *E*-value < 1e−4. Proteins which did not cluster into a subfam of at least 2 proteins were discarded from this analysis leaving a total of 964,644 (87.4%) proteins with subfamily assignments. A total of 76,216 protein subfam clusters were formed. Subsequently proteins within each subfam were aligned, HMMs were constructed from these alignments, and all-v-all HMM scoring was conducted using the hhblits.py script (which implements the hhblits function within the hhsuite v3.0 [73] software package) using default options. For HMM-HMM scoring any local alignments between HMMs of different subfams had to have an hhblits probability score of ≥95% to be retained for downstream analysis. Finally, family groupings of subfams (fams) were formed by applying the Markov clustering algorithm (MCL) [74] to the network of all HMM-HMM connections using the runningMclClustering.py script of the pipeline using the following options: runningMclClustering.py --force --min-size 2 --cpu 4 --fasta config.json. This resulted in the formation of 19,828 protein fam clusters.

### BCP identification, quantification, and phylogenetics

Cupredoxin-like BCPs were initially identified in our dataset by selecting all proteins that carried one of the following Pfam domains: Copper-bind, COX2, COX_ARM, Cu-oxidase, Cu-oxidase_2, Cu-oxidase_3, Cu_bind_like, Cupredoxin_1, CzcE, DP-EP, Ephrin, hGDE_N, PAD_N, PixA, SoxE. These domains are all members of the Pfam CU_oxidase clan (CL0026). Due to the large sequence divergence in cupredoxin-like domains (as evidenced by the number of pfam models required to appropriately capture their diversity), we posited that many divergent domain sequences would be missed by direct Pfam annotation. Alternatively, more sensitive local HMM-HMM comparison at the subfam level would cluster the majority of BCP domain containing proteins into a single fam cluster. Thus, we quantified the number of BCP domain containing proteins present in all fams. Given that 90.2% of proteins with annotated BCP domains were members of fam00018, we used fam00018 to represent BCP domain containing proteins in our dataset. Comparison of fam00018 protein counts in genomes was carried out independently for the archaeal phyla Thermoplasmatota and Thermoproteota between all archaeal orders within these phyla that contained ≥3 genomes. Global significant differences across all orders within a phylum was first tested using the Kruskal–Wallis rank sum test implemented as the kruskal.test() function in R (α ≤ 0.05). Pairwise significant differences between orders within a phylum was then tested using the Wilcoxon rank sum test implemented as the pairwise.wilcox.test() function in R. P-values from tests were corrected for multiple comparisons using FDR with a value of FDR ≤ 0.05 being considered significant. All aggregation, quantification, statistical testing, and plotting of BCP protein count data was performed using a custom R script available in our github repository.

Manual subfamily level annotation and domain architecture analysis was performed on 100 fam00018 subfams that contained at least five proteins (85.2% of all fam00018 proteins). The proteins in each subfam were re-aligned with MAFFT v7.471 using the following options: mafft --maxiterate 1000 --localpair --reorder --thread 12 [proteins in] > [alignment out]. HMMs were built for each alignment using hhsuite v3.0, and HMM-HMM scoring was performed against the PfamA database release r32 using hhsearch with the following options: hhsearch -i [input hmm] -o [output table] -d [pfam hmm database] -p 50 -E 0.001 -z 1 -Z 32000 -b 0 -B 0 -n 1 -cpu 18. Domain matches with a probability score of ≥ 95% were retained, and Pfam domains overlapping the same region on target subfam HMMs were resolved with the cath-resolve-hits function of the cath-tools package using default parameters. Each of the 100 subfams were then manually annotated and placed into 1 of 6 broad classification groups based on the subfam level Pfam domain architectures and the types of KEGG and arCOG annotations assigned to individual proteins within the subfam. Annotation groups were defined as follows: COX2 (Contains COX2 domain and KEGG or arCOG annotations indicate >40% of proteins in cluster are terminal oxidase subunits), 1D_Cu_Small (one Pfam BCP domain, mean protein length < 250 amino acids), 1D_Cu_Med (one Pfam BCP domain, mean protein length 250 - 400 amino acids), 1D_Cu_Large (one Pfam BCP domain, mean protein length > 400 amino acids), 2D_Cu (two Pfam BCP domains), 3D_Cu (three Pfam BCP domains). Due to its presence in Angelarcheales-1 and its proximity to the *coxAB* locus subfam00588 was also manually annotated as above despite having <5 proteins. Assignment of copper site types (e.g., Type 1 Copper) was conducted by manual inspection of subfamily alignments and referenced from Gräff, et al. [41]. All subfams within fam00018 and their associated annotations are available in Supplementary Table 10.

Phylogenetic tree construction for manually annotated subfams with >1 BCP domain was undertaken as follows: All proteins from subfams in the 2D_Cu and 3D_Cu manual annotation groups (*n* = 349 proteins) were combined with reference laccase and nitrite reductase (*nirK*) sequences from Decleyre, et al. [75], Kobayashi, et al. [42], and Nakamura, et al. [76] (*n* = 90 proteins). Proteins were aligned with MAFFT v7.471 using the following options: mafft --maxiterate 1000 --genafpair --reorder --thread 12 [proteins in] > [alignment out]. A maximum likelihood phylogenetic tree was constructed using IQ-TREE v1.6.12 with the following options: -m MFP -bb 1000 -alrt 1000 -nt 12. The empirically selected evolutionary rate model for the tree was WAG + F + R7 based on BIC. Branch support was estimated using ultrafast bootstrapping with 1000 bootstrap replicates. Tree was annotated and displayed using iTOL v6. Tree clades were manually defined based on positioning of reference sequences within clusters and copper site types present in sequences defined in Nakamura, et al. [76].

### Gene co-occurrence analysis and locus plotting

Contigs containing genes for the *amo*/*pmoCAXB* cluster, the *coxAB* oxygen utilizing terminal oxidase cluster, and the complex III like cytochrome b gene cluster were identified in Ca. Angelarchaeales genomes as follows: *amo*/*pmoCAXB* contigs were identified as any contig encoding an *amo*/*pmo* subunit (as described above); *coxAB* containing contigs were identified as any contig encoding a gene that matched to the KEGG HMM for K02274 (*coxA*); and complex III like gene cluster containing contigs were identified as any counting encoding a gene that matched the arCOG HMM for arCOG01721 (cytochrome b of bc complex). All loci were extracted from our master annotation table using a custom R script available in our github repository, and loci were displayed using the gggenes package in R (https://github.com/wilkox/gggenes). For ease of viewing *amo*/*pmoCAXB* and *coxAB* containing contigs were truncated to display ten genes on either side of the gene cluster of interest. Complex III containing contigs were truncated to display 22 genes on either side of the gene cluster as to allow inclusion of other proximal respiratory complexes.

### KEGG Ortholog enrichment analysis

Detection of enriched KO terms between archaeal orders was carried out using a custom R script available in our github repository. Archaeal orders from the phyla Thermoplasmatota and Thermoproteota that contained <3 genomes were excluded from the analysis. For the remainder of genomes, a table was generated giving the count of all observed KOs (*n* = 5929 total unique KO terms) in every genome. KOs that occurred <10 times across all genomes were filtered from the analysis. The filtered KO count table along with the taxonomic order level groupings of genomes were used as the input for indicator species analysis implemented as the multipatt() function in the R indicspecies package [77] using the following options: multipatt(x = [KO count matrix], cluster = [genome taxonomic assignment], func = “IndVal.g”, max.order = 2, restcomb = c(1:25, 262), control = how(nperm = 9999)). We analyzed the enrichment of KO term frequency in all orders individually as well as a grouping consisting of the combined genomes from orders Ca. Angelarcheales and Nitrosospherales. This allows for the identification of enriched functions that are shared by these two groups relative to all other lineages and to each other individually. Statistical significance of enriched KO frequency was estimated by permutation using 9,999 permuted groupings of the genomes. *P* values were corrected for multiple testing using FDR with a value of FDR ≤ 0.05 being considered significant. In addition only KOs with an indicator value ≥0.4 were retained for downstream analysis. KOs enriched in Nitrosospherales (Gp 14), Ca. Angelarcheales (Gp 17), or shared by both orders (Gp 26) were displayed as a heatmap using the superheat package in R. For a list of all significant KOs and their functions see Supplementary Table 11. Functional category assignments for KOs were derived from KEGG orthology group hierarchies and manually curated, and quantified in R. The full list of KO to functional category assignments is available in our github repository.

## Supplementary information

Combined Supplementary Figures

Combined Supplementary Figure Legends

Combined Supplementary Tables

Dataset 1

## Data Availability

Genomic data, including assembled genomes and raw sequencing reads, are available under the following NCBI BioProject accession numbers: PRJNA449266 and PRJNA288027. Additional genomes reported as part of this study can be found in NCBI BioProject PRJNA779998 and Ca. Angelarcheales genomes from the project can also be found at ggKbase (https://ggkbase.berkeley.edu/AMO_Archaea). Large datasets including the ful gene levell annotation table are available at figshare (https://figshare.com/projects/AMO_Archaea/112599).

## Code availability

Code use for the analysis in this report are available at the following github repository: https://github.com/SDmetagenomics/AMO_Archaea_2021.

## References

[1] Khadka R, Clothier L, Wang L, Lim CK, Klotz MG, Dunfield PF (2018). Evolutionary history of copper membrane monooxygenases. Front Microbiol..

[2] Coleman NV, Le NB, Ly MA, Ogawa HE, McCarl V, Wilson NL (2011). Hydrocarbon monooxygenase in Mycobacterium: recombinant expression of a member of the ammonia monooxygenase superfamily. ISME J..

[3] Lehtovirta-Morley LE (2018). Ammonia oxidation: Ecology, physiology, biochemistry and why they must all come together. FEMS Microbiol Lett..

[4] Stahl DA, de la Torre JR (2012). Physiology and diversity of ammonia-oxidizing archaea. Annu Rev Microbiol..

[5] Mancinelli RL (1995). The regulation of methane oxidation in soil. Annu Rev Microbiol..

[6] Wu L, Chen X, Wei W, Liu Y, Wang D, Ni B-J (2020). A critical review on nitrous oxide production by ammonia-oxidizing archaea. Environ Sci Technol.

[7] Monteiro M, Séneca J, Magalhães C (2014). The history of aerobic ammonia oxidizers: from the first discoveries to today. J Microbiol..

[8] Alves RJE, Minh BQ, Urich T, von Haeseler A, Schleper C (2018). Unifying the global phylogeny and environmental distribution of ammonia-oxidizing archaea based on amoA genes. Nat Commun..

[9] Knief C (2015). Diversity and habitat preferences of cultivated and uncultivated aerobic methanotrophic bacteria evaluated based on pmoA as molecular marker. Front Microbiol.

[10] Hatzenpichler R (2012). Diversity, physiology, and niche differentiation of ammonia-oxidizing archaea. Appl Environ Microbiol.

[11] Leininger S, Urich T, Schloter M, Schwark L, Qi J, Nicol GW (2006). Archaea predominate among ammonia-oxidizing prokaryotes in soils. Nature..

[12] Liu S, Hu B, He Z, Zhang B, Tian G, Zheng P (2015). Ammonia-oxidizing archaea have better adaptability in oxygenated/oypoxic alternant conditions compared to ammonia-oxidizing bacteria. Appl Microbiol Biotechnol..

[13] Tolar BB, Herrmann J, Bargar JR, van den Bedem H, Wakatsuki S, Francis CA (2017). Integrated structural biology and molecular ecology of N-cycling enzymes from ammonia-oxidizing archaea. Environ Microbiol Rep..

[14] Bédard C, Knowles R (1989). Physiology, biochemistry, and specific inhibitors of CH4, NH4+, and CO oxidation by methanotrophs and nitrifiers. Microbiol Rev.

[15] Burrows KJ, Cornish A, Scott D, Higgins IJ (1984). Substrate specificities of the soluble and particulate methane mono-oxygenases of Methylosinus trichosporium OB3b. Microbiology.

[16] Qin W, Zheng Y, Zhao F, Wang Y, Urakawa H, Martens-Habbena W (2020). Alternative strategies of nutrient acquisition and energy conservation map to the biogeography of marine ammonia-oxidizing archaea. ISME J.

[17] Lancaster KM, Caranto JD, Majer SH, Smith MA (2018). Alternative bioenergy: updates to and challenges in nitrification metalloenzymology. Joule.

[18] Vajrala N, Martens-Habbena W, Sayavedra-Soto LA, Schauer A, Bottomley PJ, Stahl DA (2013). Hydroxylamine as an intermediate in ammonia oxidation by globally abundant marine archaea. Proc Natl Acad Sci.

[19] Hosseinzadeh P, Tian S, Marshall NM, Hemp J, Mullen T, Nilges MJ (2016). A purple cupredoxin from Nitrosopumilus maritimus containing a mononuclear type 1 copper center with an open binding site. J Am Chem Soc..

[20] Stein LY (2019). Insights into the physiology of ammonia-oxidizing microorganisms. Curr Opin Chem Biol.

[21] Carini P, Dupont CL, Santoro AE (2018). Patterns of thaumarchaeal gene expression in culture and diverse marine environments. Environ Microbiol..

[22] Sirajuddin S, Rosenzweig AC (2015). Enzymatic oxidation of methane. Biochemistry..

[23] Chistoserdova L, Vorholt JA, Lidstrom ME (2005). A genomic view of methane oxidation by aerobic bacteria and anaerobic archaea. Genome Biol..

[24] Pester M, Rattei T, Flechl S, Gröngröft A, Richter A, Overmann J (2012). AmoA-based consensus phylogeny of ammonia-oxidizing archaea and deep sequencing of amoA genes from soils of four different geographic regions. Environ Microbiol..

[25] Diamond S, Andeer PF, Li Z, Crits-Christoph A, Burstein D, Anantharaman K (2019). Mediterranean grassland soil C-N compound turnover is dependent on rainfall and depth, and is mediated by genomically divergent microorganisms. Nat Microbiol.

[26] Sharrar AM, Crits-Christoph A, Méheust R, Diamond S, Starr EP, Banfield JF (2020). Bacterial secondary metabolite biosynthetic potential in soil varies with phylum, depth, and vegetation type. mBio.

[27] Lavy A, McGrath DG, Carnevali PBM, Wan J, Dong W, Tokunaga TK (2019). Microbial communities across a hillslope‐riparian transect shaped by proximity to the stream, groundwater table, and weathered bedrock. Ecol Evol.

[28] Anantharaman K, Brown CT, Hug LA, Sharon I, Castelle CJ, Probst AJ (2016). Thousands of microbial genomes shed light on interconnected biogeochemical processes in an aquifer system. Nat Commun.

[29] Dong X, Greening C, Rattray JE, Chakraborty A, Chuvochina M, Mayumi D (2019). Metabolic potential of uncultured bacteria and archaea associated with petroleum seepage in deep-sea sediments. Nat Commun.

[30] Meinhardt KA, Bertagnolli A, Pannu MW, Strand SE, Brown SL, Stahl DA (2015). Evaluation of revised polymerase chain reaction primers for more inclusive quantification of ammonia-oxidizing archaea and bacteria. Environ Microbiol Rep..

[31] Wang VCC, Maji S, Chen PPY, Lee HK, Yu SSF, Chan SI (2017). Alkane oxidation: methane monooxygenases, related enzymes, and their biomimetics. Chem Rev..

[32] Lawton TJ, Ham J, Sun T, Rosenzweig AC (2014). Structural conservation of the B subunit in the ammonia monooxygenase/particulate methane monooxygenase superfamily. Proteins.

[33] Jumper J, Evans R, Pritzel A, Green T, Figurnov M, Ronneberger O (2021). Highly accurate protein structure prediction with AlphaFold. Nature.

[34] Rinke C, Chuvochina M, Mussig AJ, Chaumeil P-A, Davín AA, Waite DW (2021). A standardized archaeal taxonomy for the Genome Taxonomy Database. Nat Microbiol..

[35] Chain PSG, Grafham DV, Fulton RS, FitzGerald MG, Hostetler J, Muzny D (2009). Genome project standards in a new era of sequencing. Science.

[36] Zinke LA, Evans PN, Santos‐Medellín C, Schroeder AL, Parks DH, Varner RK (2021). Evidence for non‐methanogenic metabolisms in globally distributed archaeal clades basal to the Methanomassiliicoccales. Environ Microbiol.

[37] Zhou Z, Pan J, Wang F, Gu J-D, Li M (2018). Bathyarchaeota: globally distributed metabolic generalists in anoxic environments. FEMS Microbiol Rev..

[38] Méheust R, Burstein D, Castelle CJ, Banfield JF (2019). The distinction of CPR bacteria from other bacteria based on protein family content. Nat Commun..

[39] Reji L, Francis CA (2020). Metagenome-assembled genomes reveal unique metabolic adaptations of a basal marine Thaumarchaeota lineage. ISME J..

[40] Komori H, Higuchi Y (2010). Structure and molecular evolution of multicopper blue proteins. Biomol Concepts.

[41] Gräff M, Buchholz PCF, Roes‐Hill ML, Pleiss J (2020). Multicopper oxidases: modular structure, sequence space, and evolutionary relationships. Proteins Struct Funct Bioinforma..

[42] Kobayashi S, Hira D, Yoshida K, Toyofuku M, Shida Y, Ogasawara W (2018). Nitric oxide production from nitrite reduction and hydroxylamine oxidation by copper-containing dissimilatory nitrite reductase (nirK) from the aerobic ammonia-oxidizing archaeon, Nitrososphaera viennensis. Microbes Environ.

[43] Reyes C, Hodgskiss LH, Kerou M, Pribasnig T, Abby SS, Bayer B (2020). Genome wide transcriptomic analysis of the soil ammonia oxidizing archaeon Nitrososphaera viennensis upon exposure to copper limitation. ISME J..

[44] Kitzinger K, Padilla CC, Marchant HK, Hach PF, Herbold CW, Kidane AT (2019). Cyanate and urea are substrates for nitrification by thaumarchaeota in the marine environment. Nat Microbiol.

[45] Bräsen C, Esser D, Rauch B, Siebers B (2014). Carbohydrate metabolism in archaea: current insights into unusual enzymes and pathways and their regulation. Microbiol Mol Biol Rev..

[46] Tjaden B, Plagens A, Dörr C, Siebers B, Hensel R (2006). Phosphoenolpyruvate synthetase and pyruvate, phosphate dikinase of Thermoproteus tenax: key pieces in the puzzle of archaeal carbohydrate metabolism. Mol Microbiol..

[47] Chadwick GL, Hemp J, Fischer WW, Orphan VJ (2018). Convergent evolution of unusual complex I homologs with increased proton pumping capacity: energetic and ecological implications. ISME J..

[48] Welte C, Deppenmeier U (2011). Membrane-bound electron transport in Methanosaeta thermophila. J Bacteriol..

[49] Butterfield CN, Li Z, Andeer PF, Spaulding S, Thomas BC, Singh A (2016). Proteogenomic analyses indicate bacterial methylotrophy and archaeal heterotrophy are prevalent below the grass root zone. PeerJ.

[50] Yabusaki SB, Wilkins MJ, Fang Y, Williams KH, Arora B, Bargar J (2017). Water table dynamics and biogeochemical cycling in a shallow, variably-saturated floodplain. Environ Sci Technol.

[51] Ward LM, Johnston DT, Shih PM (2021). Phanerozoic radiation of ammonia oxidizing bacteria. Sci Rep..

[52] Eddy SR (2011). Accelerated profile HMM searches. PLoS Computational Biol..

[53] Parks DH, Chuvochina M, Chaumeil P-A, Rinke C, Mussig AJ, Hugenholtz P (2020). A complete domain-to-species taxonomy for bacteria and archaea. Nat Biotechnol.

[54] Chaumeil P-A, Mussig AJ, Hugenholtz P, Parks DH (2019). GTDB-Tk: a toolkit to classify genomes with the Genome Taxonomy Database. Bioinformatics..

[55] Olm MR, Brown CT, Brooks B, Banfield JF (2017). dRep: a tool for fast and accurate genomic comparisons that enables improved genome recovery from metagenomes through de-replication. ISME J..

[56] Parks DH, Imelfort M, Skennerton CT, Hugenholtz P, Tyson GW (2015). CheckM: assessing the quality of microbial genomes recovered from isolates, single cells, and metagenomes. Genome Res.

[57] Sela I, Ashkenazy H, Katoh K, Pupko T (2015). GUIDANCE2: accurate detection of unreliable alignment regions accounting for the uncertainty of multiple parameters. Nucleic Acids Res..

[58] Gibrat J-F, Madej T, Bryant SH (1996). Surprising similarities in structure comparison. Curr Opin Struct Biol..

[59] Li Z, Jaroszewski L, Iyer M, Sedova M, Godzik A (2020). FATCAT 2.0: towards a better understanding of the structural diversity of proteins. Nucleic Acids Res.

[60] Nguyen L-T, Schmidt HA, von Haeseler A, Minh BQ (2014). IQ-TREE: a fast and effective stochastic algorithm for estimating maximum-likelihood phylogenies. Mol Biol Evol..

[61] Lee MD (2019). GToTree: a user-friendly workflow for phylogenomics. Bioinformatics.

[62] Edgar RC (2004). MUSCLE: multiple sequence alignment with high accuracy and high throughput. Nucleic Acids Res..

[63] Capella-Gutiérrez S, Silla-Martínez JM, Gabaldón T (2009). trimAl: a tool for automated alignment trimming in large-scale phylogenetic analyses. Bioinformatics.

[64] Boyd JA, Woodcroft BJ, Tyson GW (2018). GraftM: a tool for scalable, phylogenetically informed classification of genes within metagenomes. Nucleic Acids Res..

[65] Wickham H (eds). ggplot2: elegant graphics for data analysis. 2nd ed. Heidelberg:Springer Science & Business Media;2009.

[66] Quinn TP, Richardson MF, Lovell D, Crowley TM (2017). propr: an R-package for identifying proportionally abundant features using compositional data analysis. Sci Rep..

[67] Quinn TP, Erb I, Gloor G, Notredame C, Richardson MF, Crowley TM (2019). A field guide for the compositional analysis of any-omics data. Gigascience.

[68] Hyatt D, Chen G-L, LoCascio PF, Land ML, Larimer FW, Hauser LJ (2010). Prodigal: prokaryotic gene recognition and translation initiation site identification. BMC Bioinforma.

[69] Aramaki T, Blanc-Mathieu R, Endo H, Ohkubo K, Kanehisa M, Goto S (2019). KofamKOALA: KEGG ortholog assignment based on profile HMM and adaptive score threshold. Bioinformatics.

[70] Huerta-Cepas J, Szklarczyk D, Heller D, Hernández-Plaza A, Forslund SK, Cook H (2018). eggNOG 5.0: a hierarchical, functionally and phylogenetically annotated orthology resource based on 5090 organisms and 2502 viruses. Nucleic Acids Res..

[71] Mistry J, Chuguransky S, Williams L, Qureshi M, Salazar GA, Sonnhammer ELL (2020). Pfam: The protein families database in 2021. Nucleic Acids Res..

[72] Steinegger M, Söding J (2017). MMseqs2 enables sensitive protein sequence searching for the analysis of massive data sets. Nat Biotechnol..

[73] Steinegger M, Meier M, Mirdita M, Vöhringer H, Haunsberger SJ, Söding J (2019). HH-suite3 for fast remote homology detection and deep protein annotation. BMC Bioinforma..

[74] Enright AJ, Dongen SV, Ouzounis CA (2002). An efficient algorithm for large-scale detection of protein families. Nucleic Acids Res..

[75] Helen D, Kim H, Tytgat B, Anne W (2016). Highly diverse nirK genes comprise two major clades that harbor ammonium-producing denitrifiers. BMC Genom..

[76] Nakamura K, Go N (2005). Function and molecular evolution of multicopper blue proteins. Cell Mol Life Sci.

[77] Cáceres MD, Legendre P (2009). Associations between species and groups of sites: indices and statistical inference. Ecology.

