# Supplementary material for: Soils and sediments host Thermoplasmata archaea encoding novel copper membrane monooxygenases (CuMMOs)
Source: ISME J. 2022 Jan 5;16(5):1348–62. doi: 10.1038/s41396-021-01177-5 (PMC9038741; doi:10.1038/s41396-021-01177-5)

Supplementary Figure 1

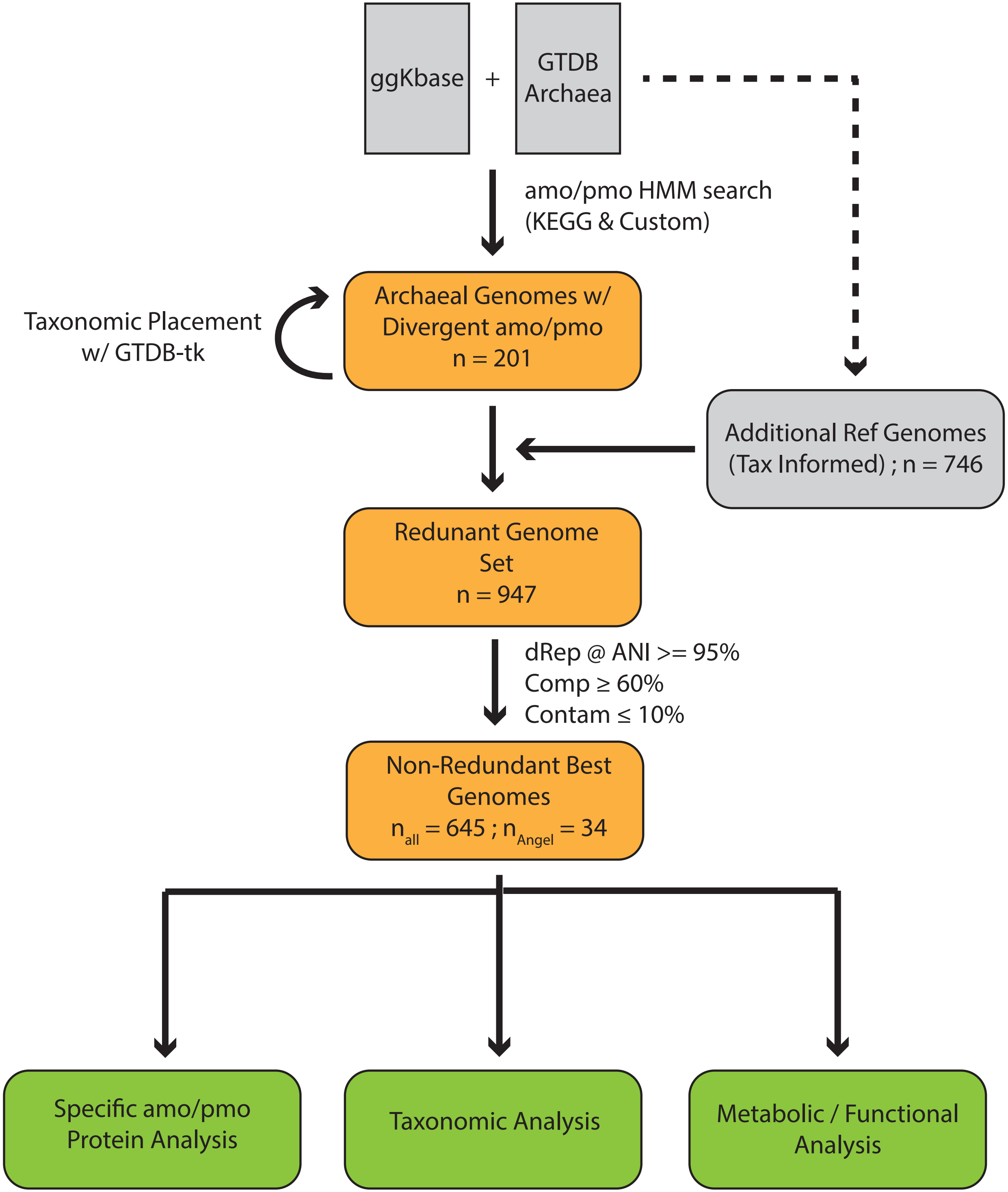

Supplementary Figure 2

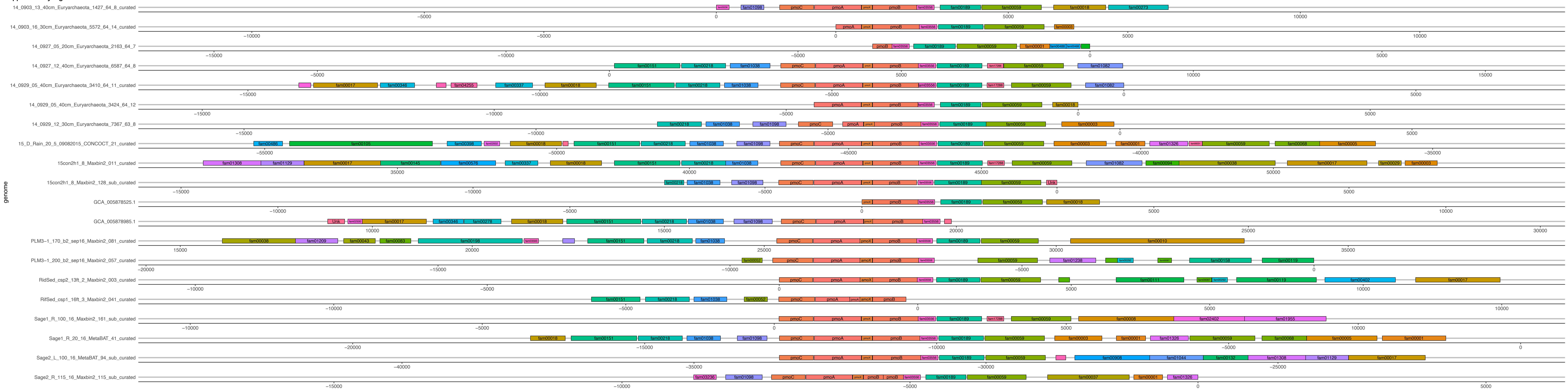

Supplementary Figure 3

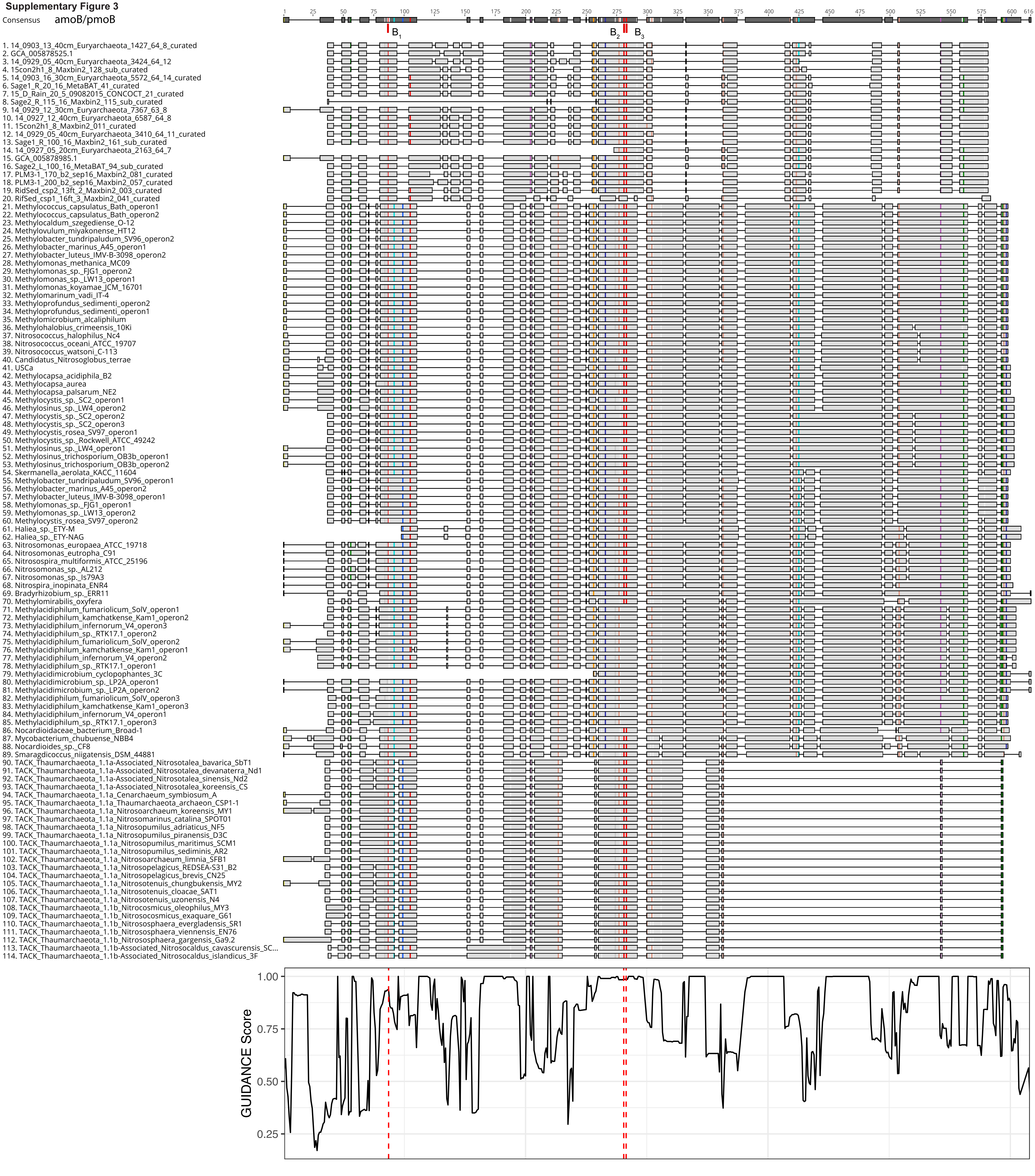

Supplementary Figure 4

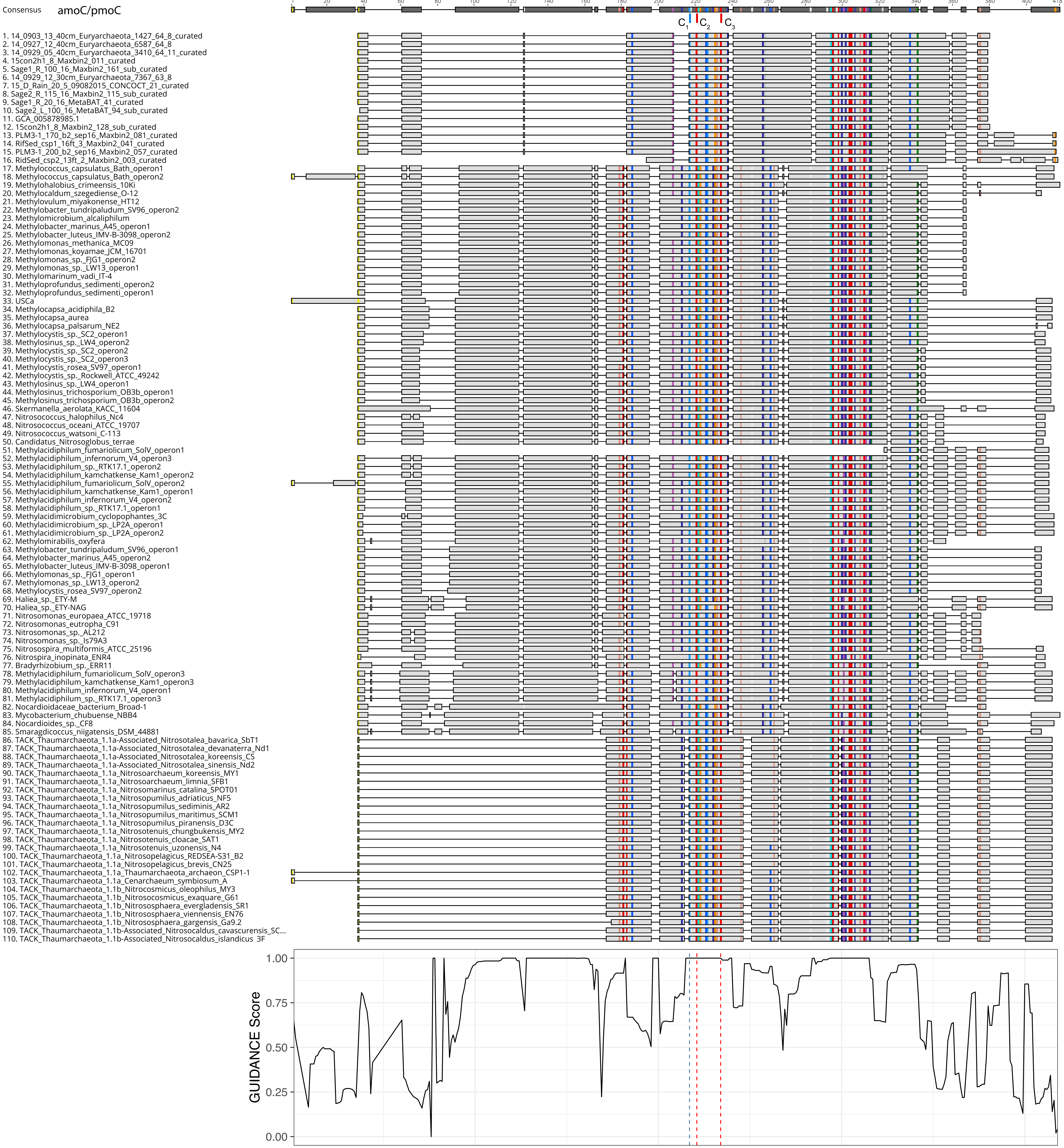

Supplementary Figure 5

A

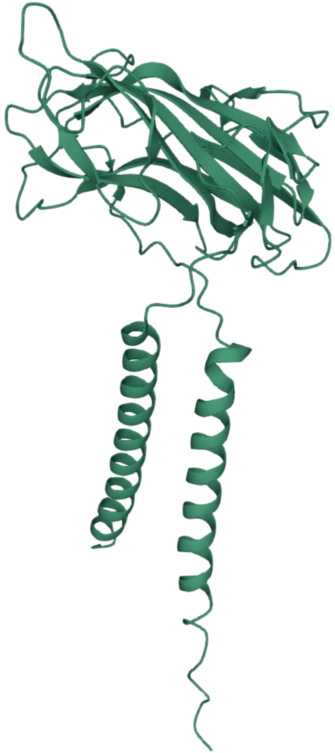

Novel amoB/pmoB
(Angelarchaeales-1)

B

Structure ID	Description	Sequence Length	Modeled Residues	Coverage
Novel amoB/pmoB	N/A	N/A	286	42%
4O65.A	Putative archaeal ammonia monooxygenase subunit B	166	153	78%

Select View   Download Files

ALIGNMENT

RMSD	TM-score	Score	SI%	SS%	Length
3.06	0.36	202.9	15	32	120

```
50   EVAYANIRVSSAPIYAHYA----------------------TQTLPLTGS   77
6    QLQSRFVKIEDETFSATRLITKAEFAKRTFGNSDLETLKQVDAMGCDPAR   55
78   GTPTTAVMGTQGYPTISLNTGEFLVVTWTFNYVATNAVRGDLFWITRLGL  127
56   R-------------------QDVLIVTGRLVSQVKQDLNAWISLFT---N   89
128  DFATIGRYITCTLPAPEPATADCTGTNTWIGSSSFLLVPGARYDATLVLR  177
90   RWEFISRDP-------------------PG--NVFTIPGGGEVPYKLCLS  118
178  SIQPTFRHLHVGVSVQNVGNIPAAPGPAGVTGNPPIPVLYGAFTDANGPV  227
119  ALEPGTYHAHTQLNIASVGPG----------------LGPGMSIVVEGEP  152
228  VEPL                                               231
153  TEKP                                               156
```

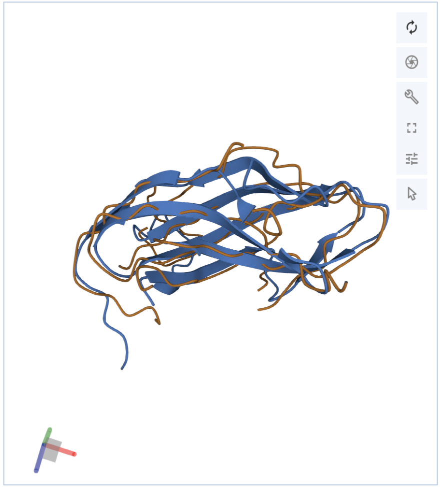

Supplementary Figure 6

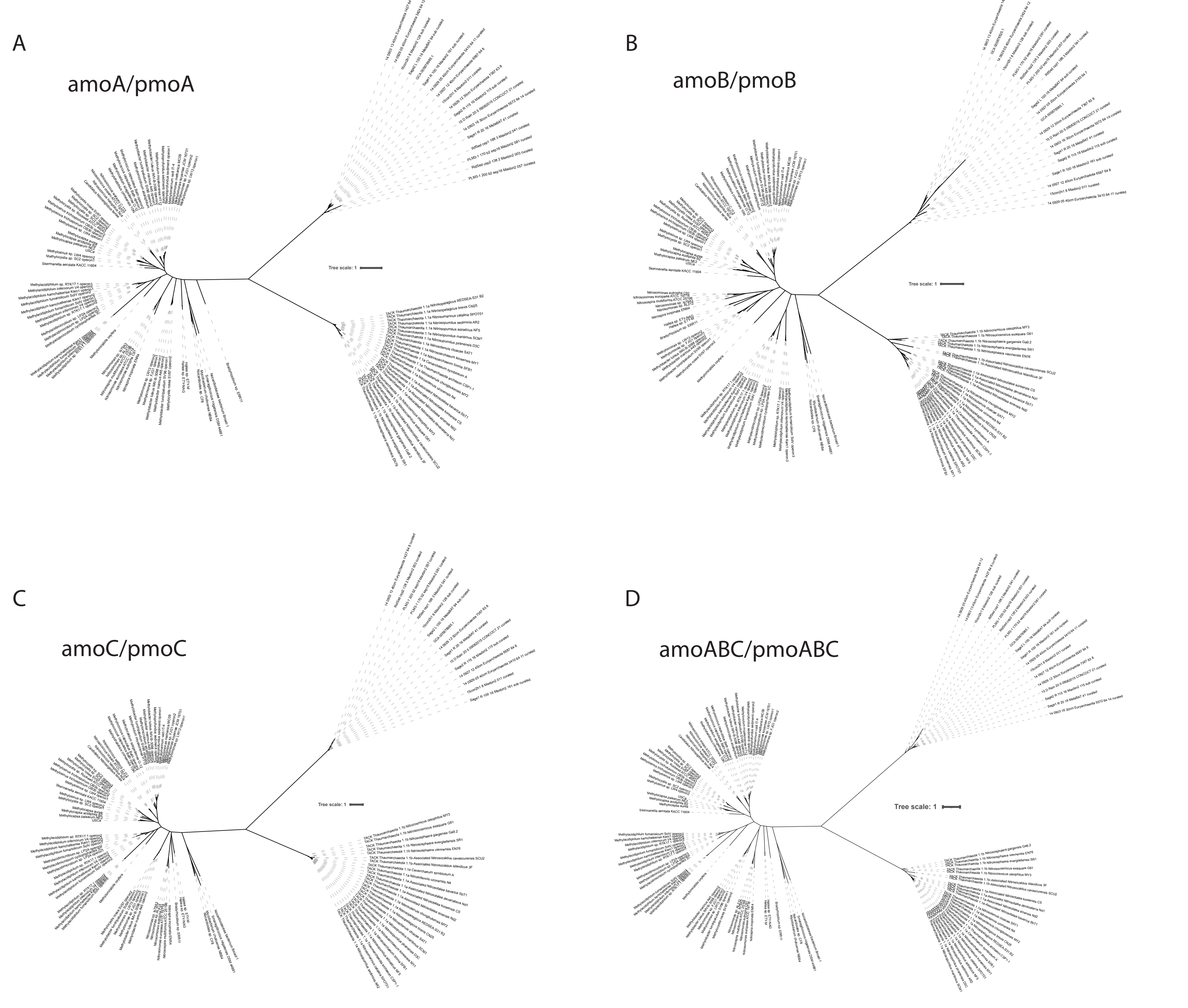

Supplementary Figure 7

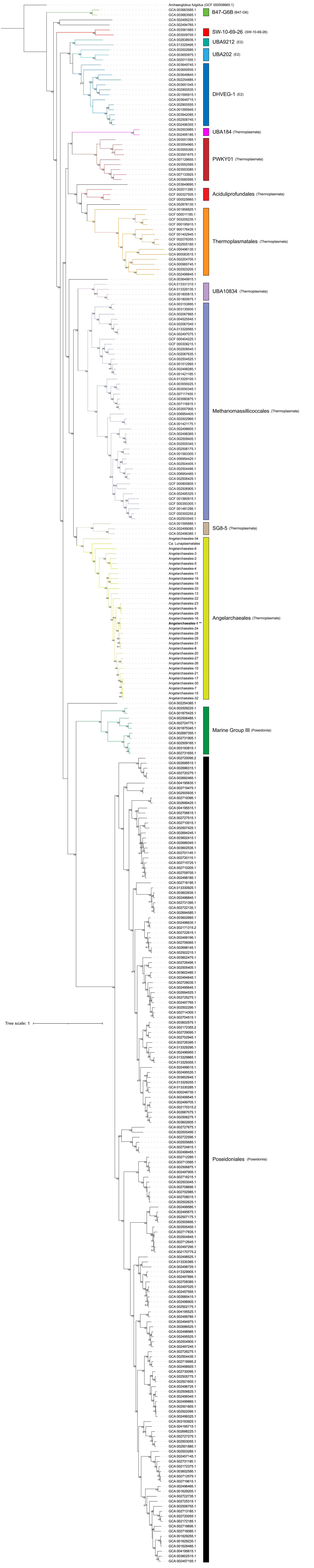

Supplementary Figure 8

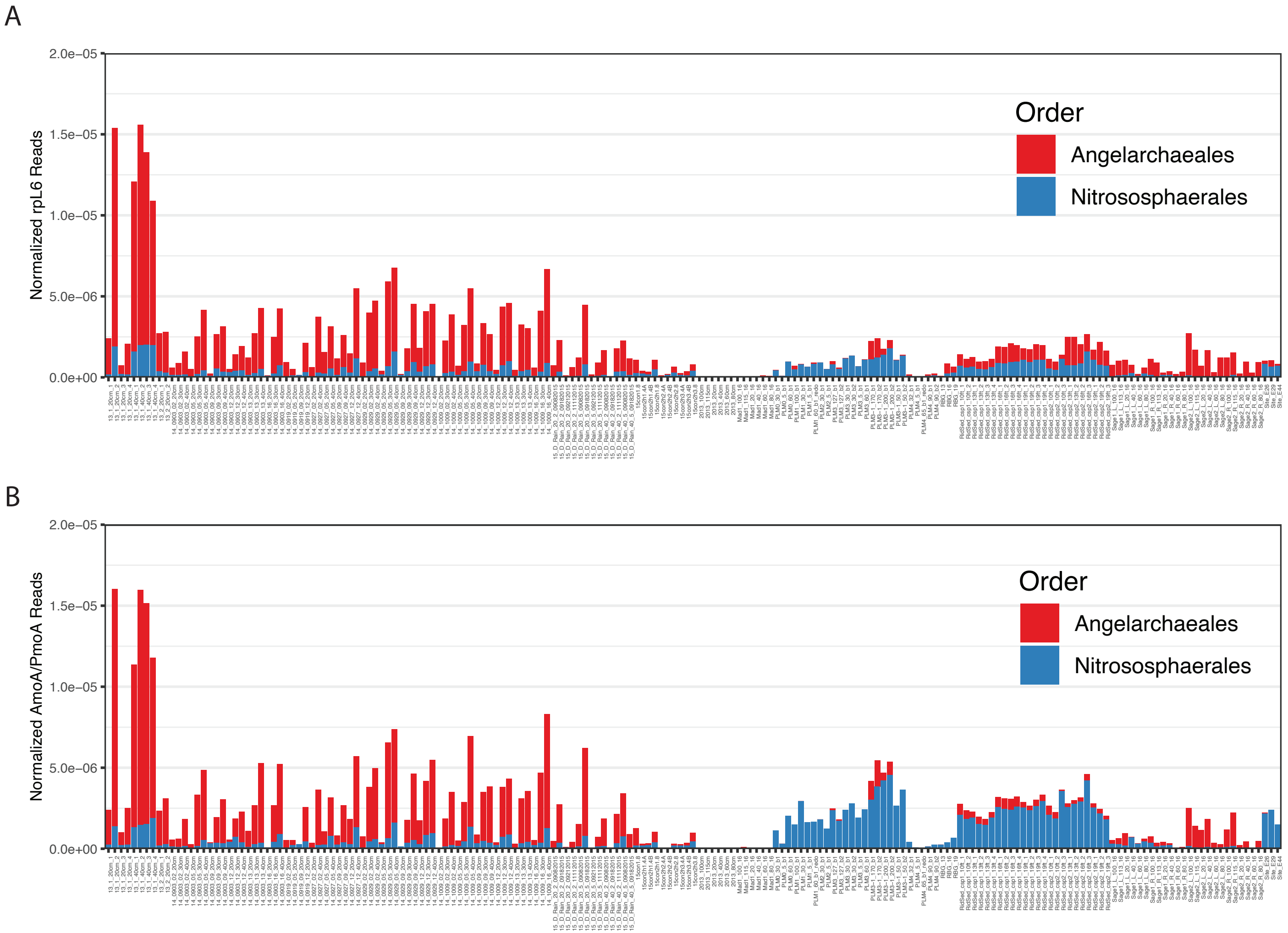

Supplementary Figure 9

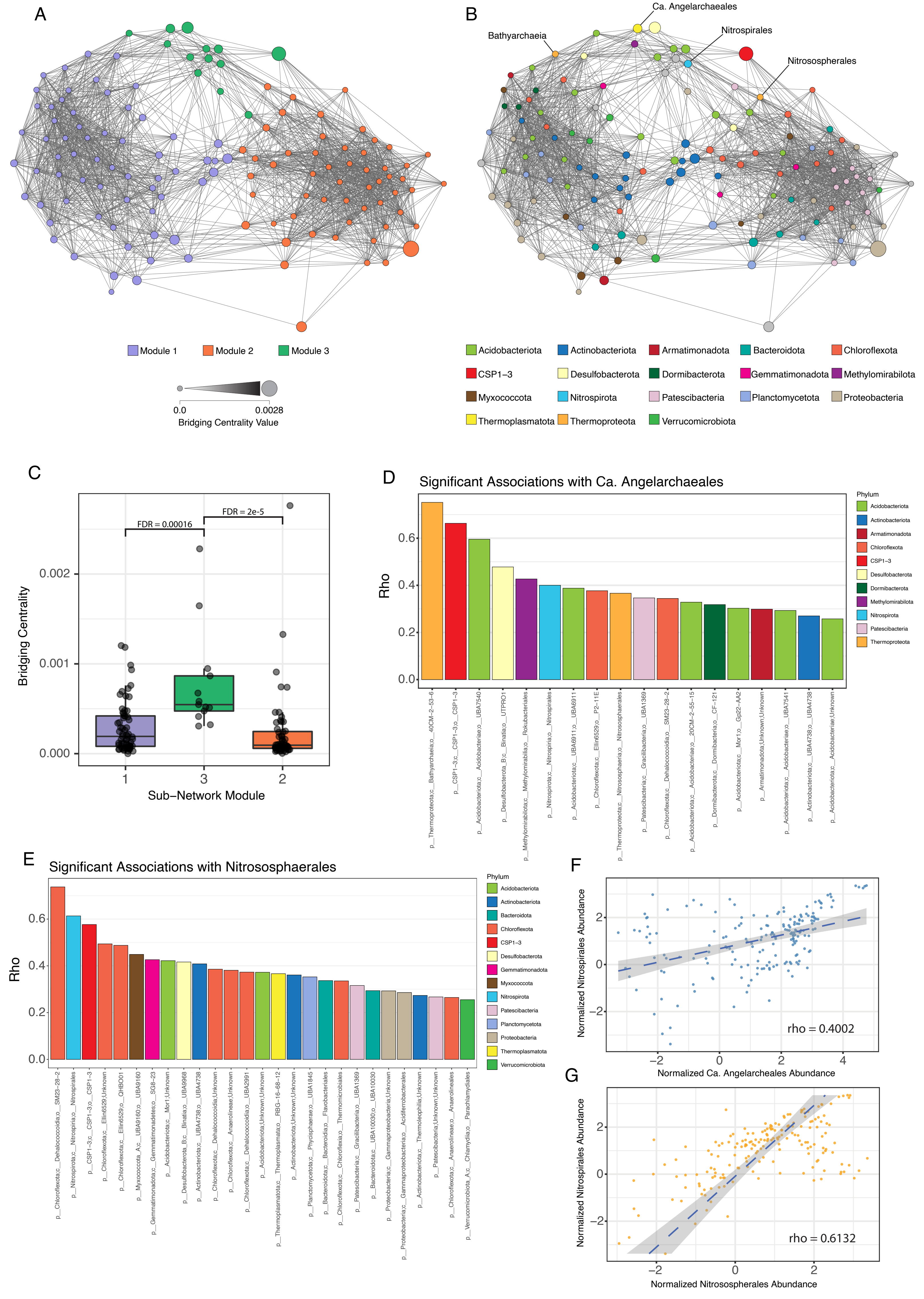

Supplementary Figure 10

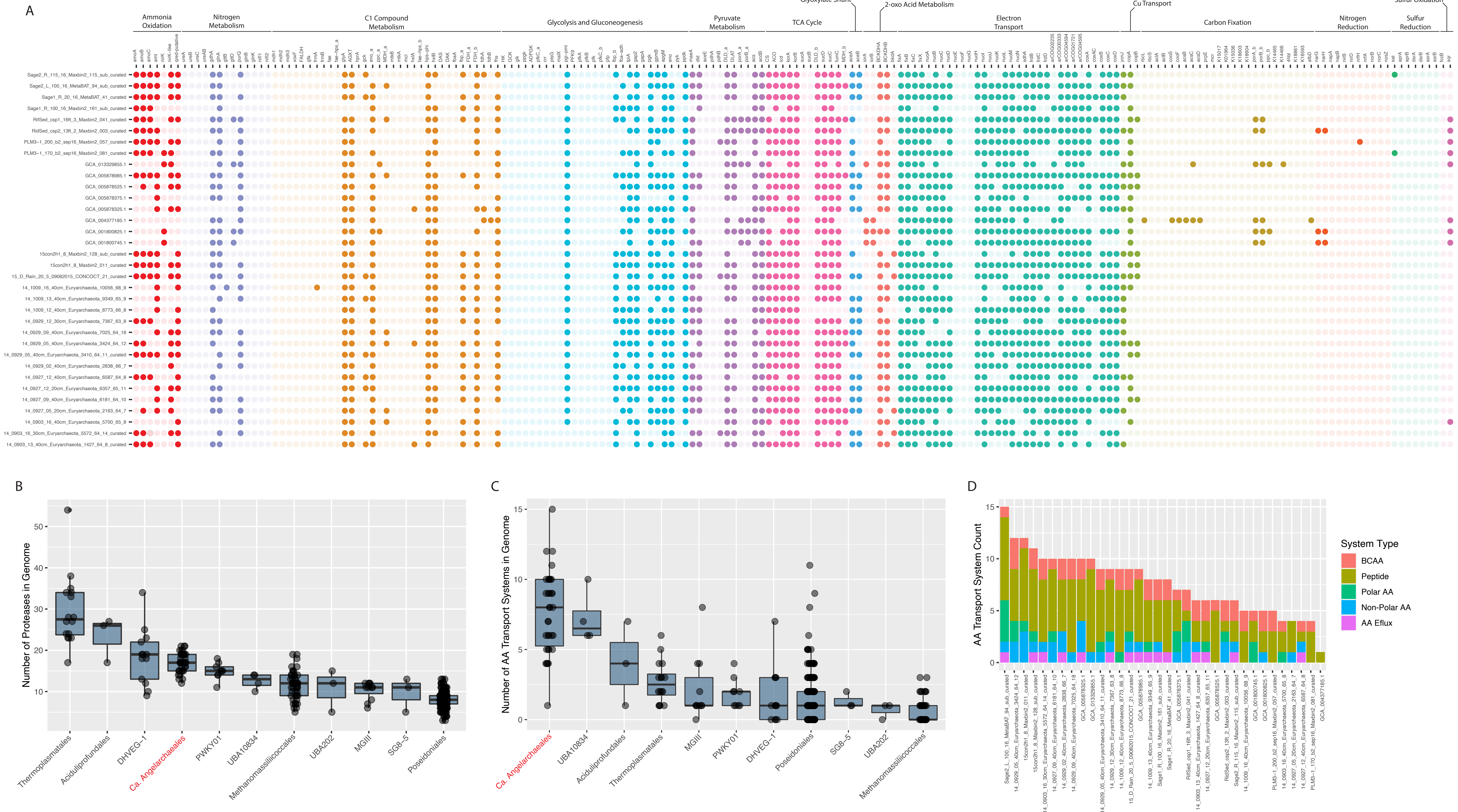

Supplementary Figure 11

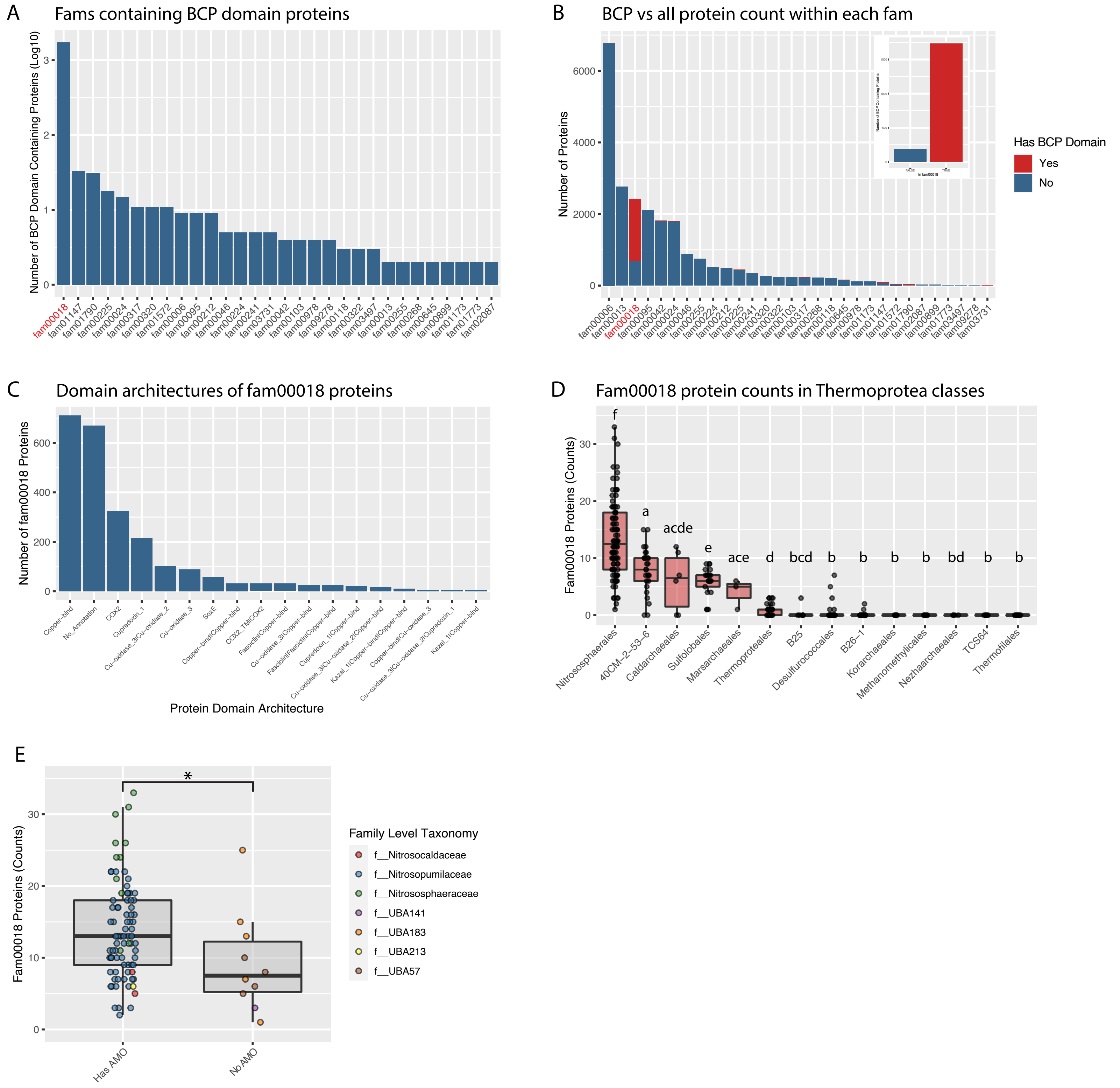

Supplementary Figure 12

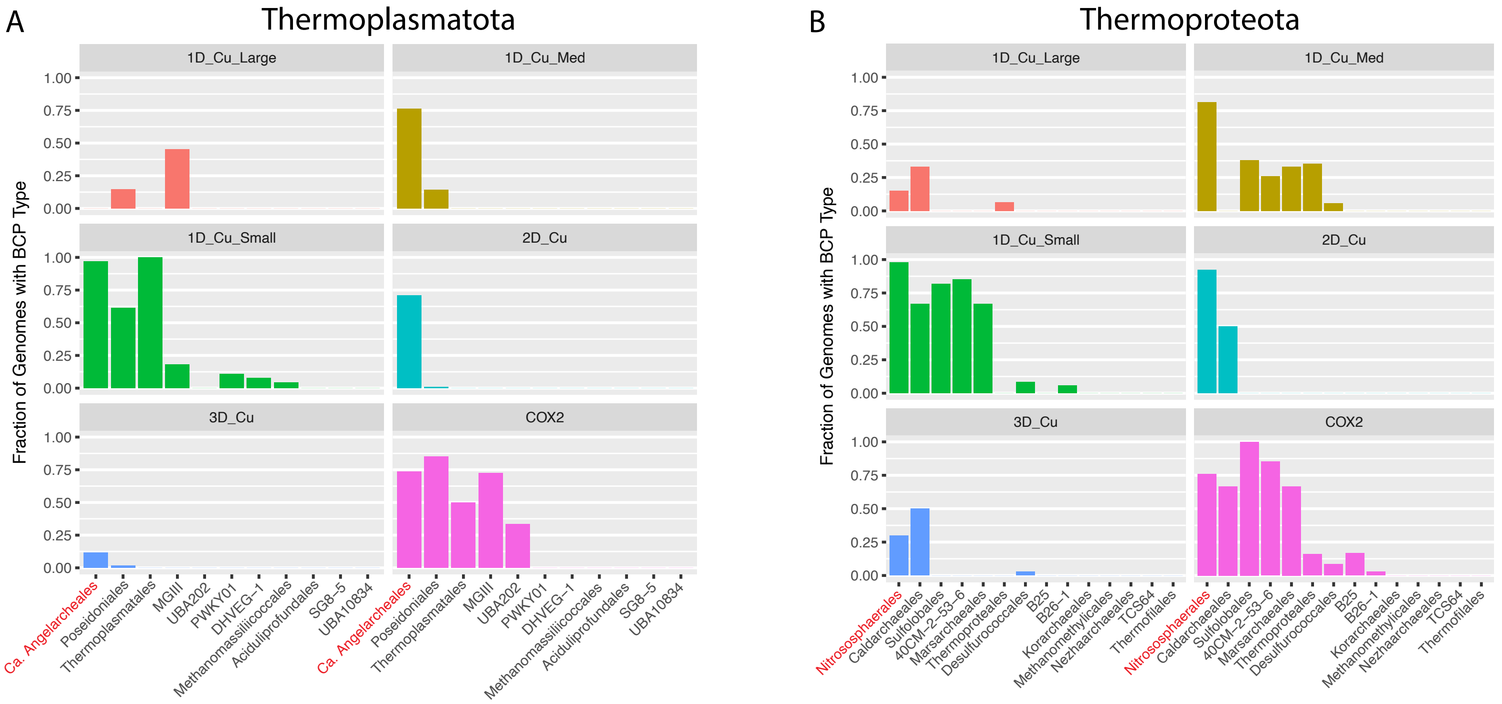

Supplementary Figure 13

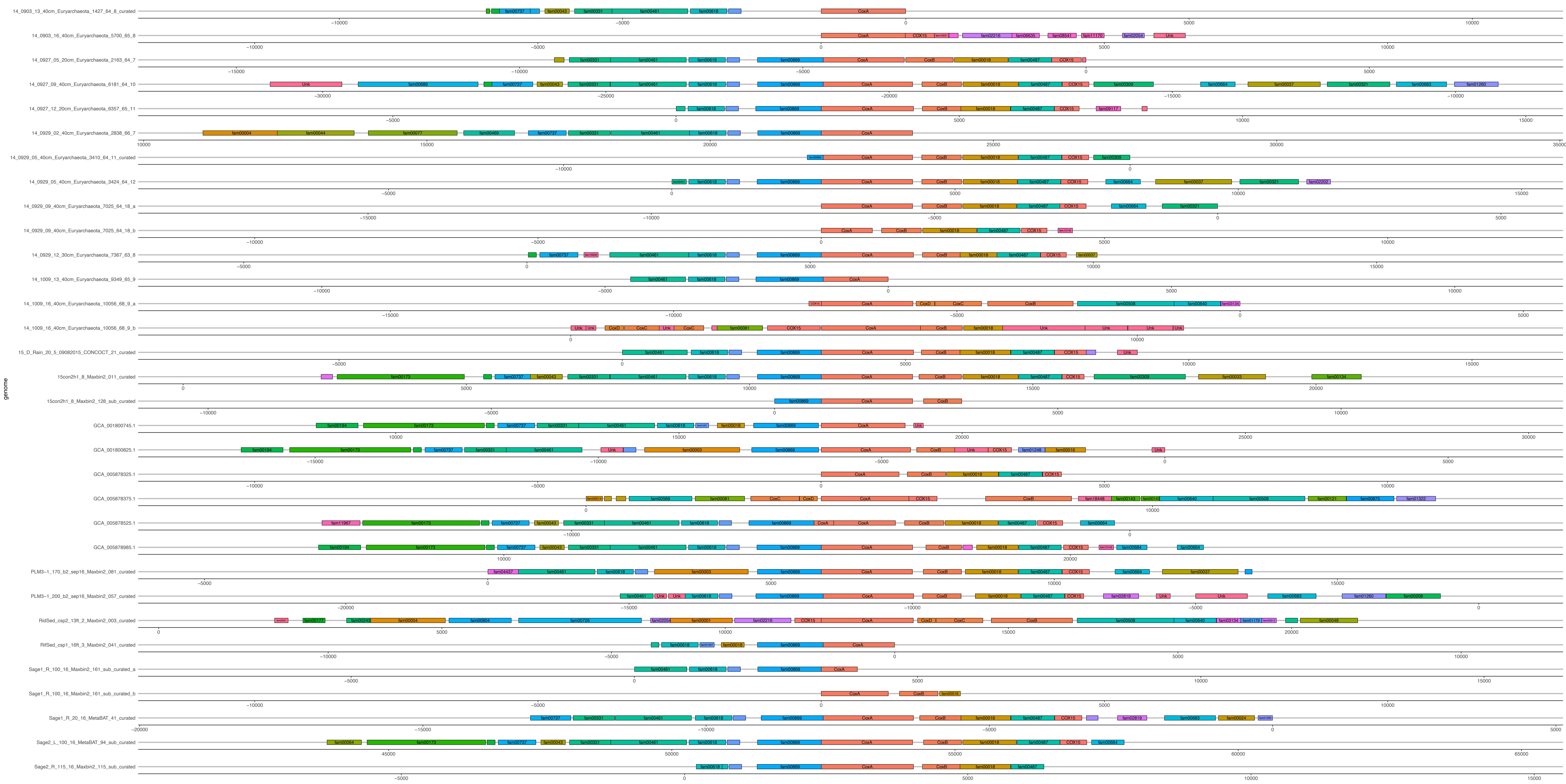

Supplementary Figure 14

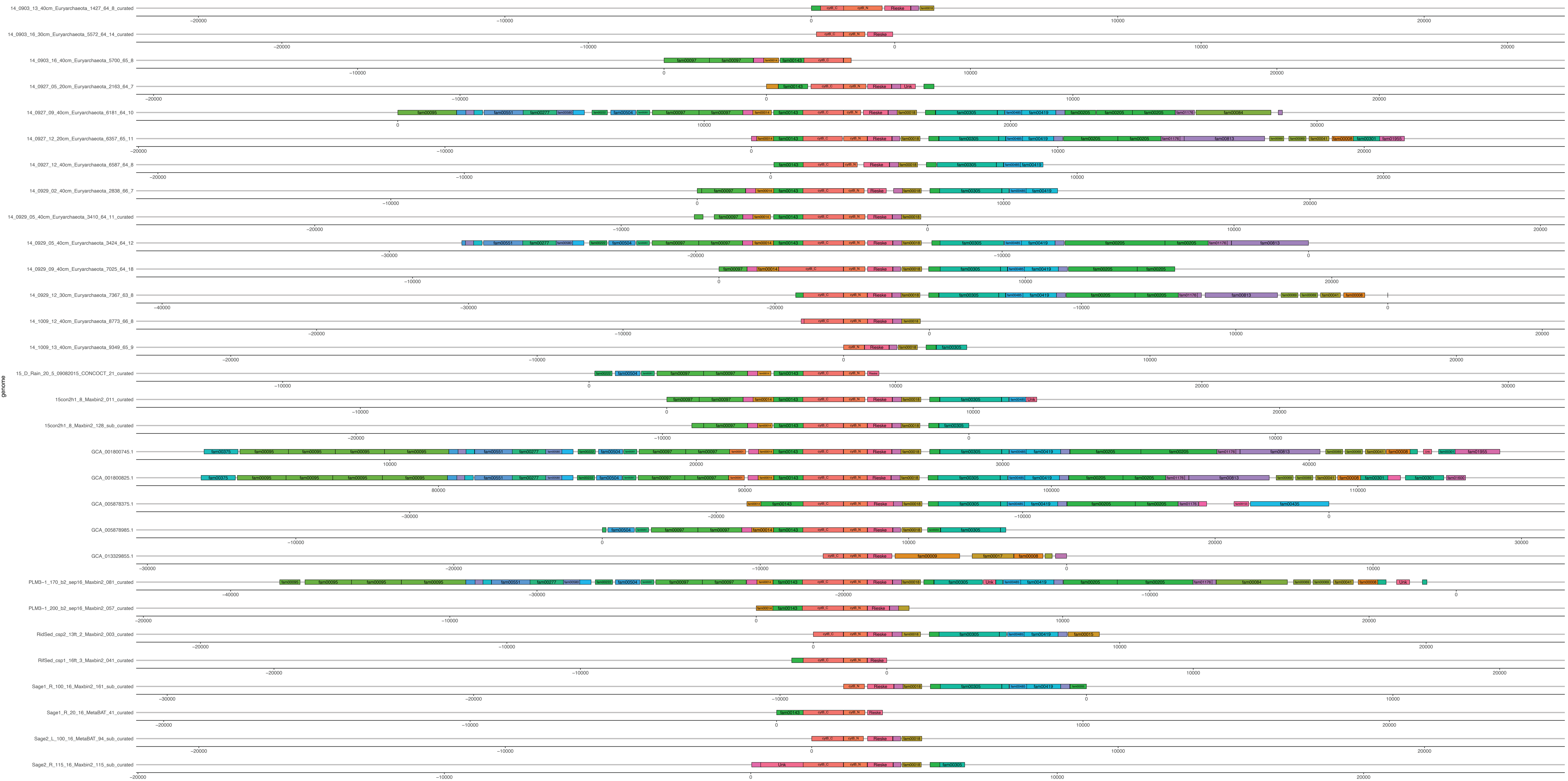

Supplementary Figure 15

TMHMM Analysis for Proteins of Subfam17112

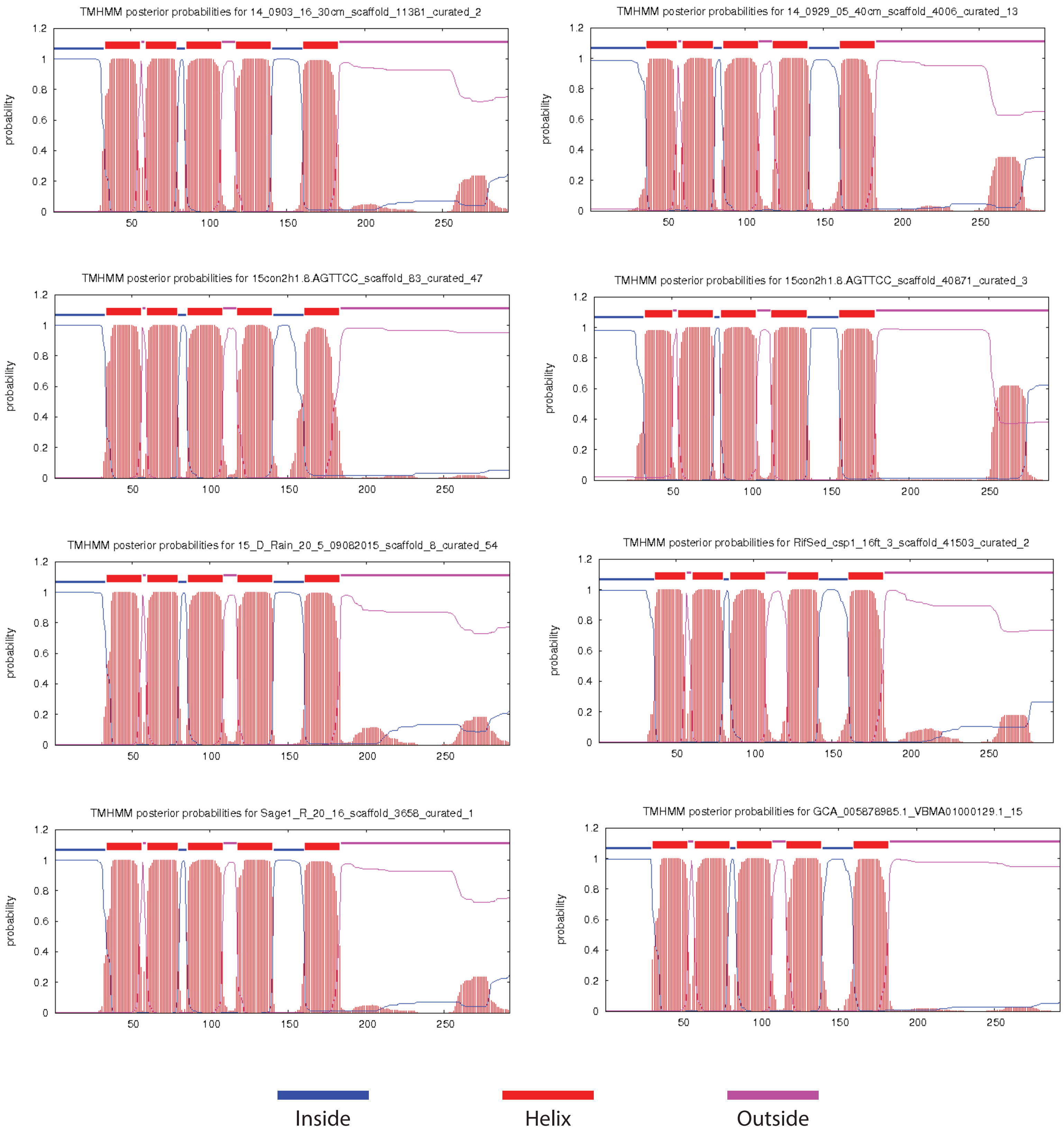

Supplement: Supplementary file 1 — Combined Supplementary Figures [file 41396_2021_1177_MOESM1_ESM.pdf]
